# Enhancing automic and optimal control systems through graphical structures

**DOI:** 10.1038/s41598-024-53244-4

**Published:** 2024-02-07

**Authors:** Sumati Kumari Panda, Ilyas Khan, Vijayakumar Velusamy, Shafiullah Niazai

**Affiliations:** 1 Department of Mathematics, GMR Institute of Technology, Rajam, Andhra Pradesh 532127 India; 2 Department of Mathematics, College of Science Al-Zulfi, Majmaah University, 11952 Al-Majmaah, Saudi Arabia; 3 Department of Mathematics, School of Advanced Sciences, Vellore Institute of Technology, Vellore, Tamil Nadu 632 014 India; 4 Department of Mathematics, Education Faculty, Laghman University, Mehtarlam City, Laghman 2701 Afghanistan

**Keywords:** Applied mathematics, Pure mathematics

## Abstract

The concept of graphical structures of extended suprametric space is introduced in this study and applied to supra-graphical contractive mapping. A recursive algorithm in connection with graphical notions can be employed in adaptive systems to construct a desired output function iteratively after specific conditions are first defined to ensure the existence of the solution by use of supra-graphical contractive mapping. After analyzing the historical context and relevant outcomes, we discuss the usage of graphical structures and supra-graphical contractive mappings in the conceptual frameworks of adaptive control and optimal control systems.

## Introduction

A graph is defined as a mathematical framework that connects a collection of points to express a specific function. A pair-wise connection among the objects is established using it. The edges that link the vertices (also known as nodes) of the graph are called vertices. Theoretically referred to as ${\mathscr {G}}={\mathscr {G}}({\mathscr {V}},{\mathcal {E}})$. Since graphs are essentially a method of encoding data, each characteristic of a graph corresponds to an actual component or notion in the information being represented. In order to fully appreciate graphs’ articulation and universality as an embedding method, it is important to be familiar with how they can be utilized for illustrating complexities.

**Figure 1:**
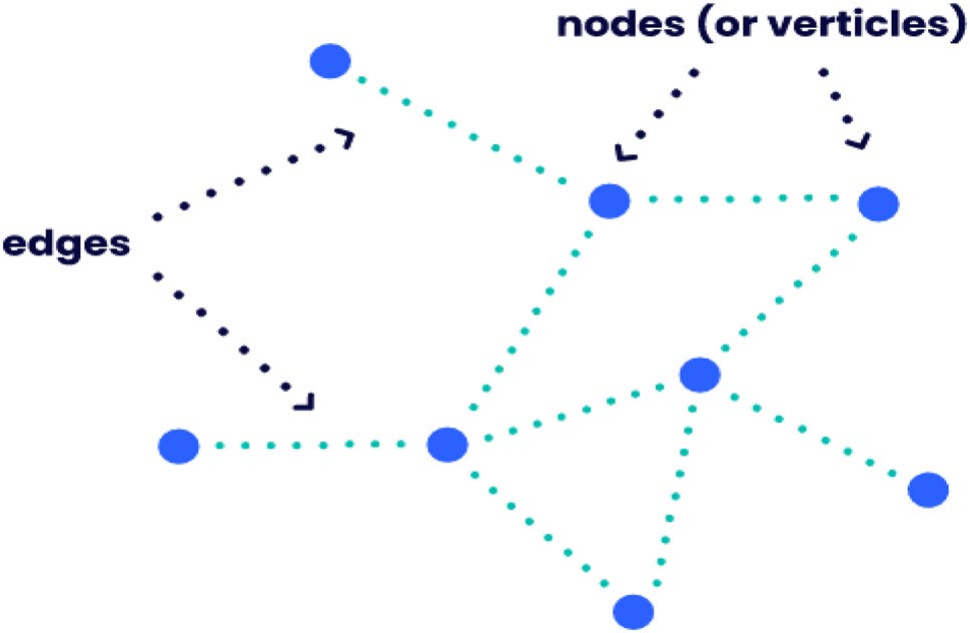
A plot that depicts the vertices and edges.

**Figure 2:**
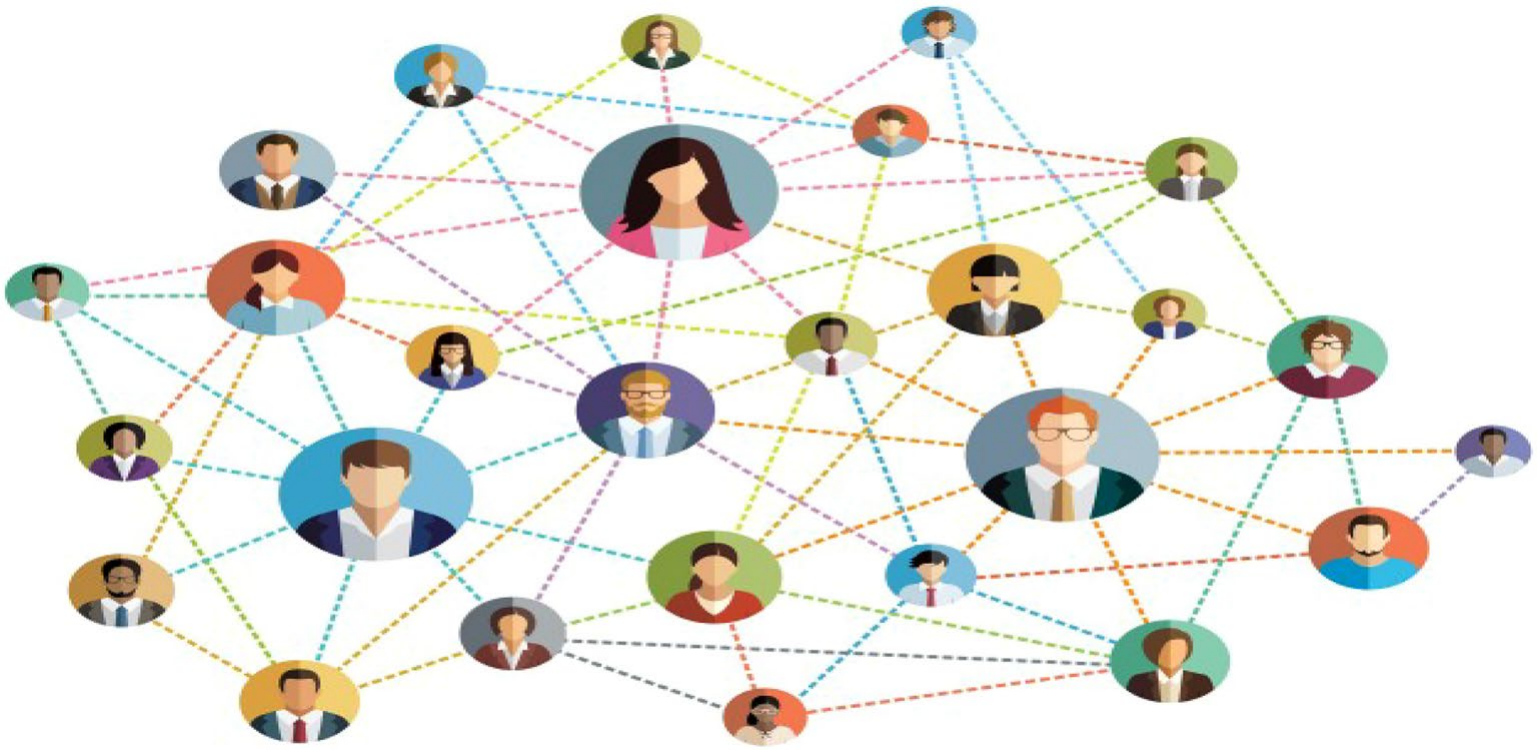
A sample graph represents connections in the online community.

Figure 1 depicts the vertices and edges. A phenomenon according to the investigation has attributes, which are measurable characteristics. Attributes can be utilized to elaborate on both edges and vertices in the graph environment. We may have attributes for every individual (vertex) that quantify their current age, recognition, and online activity, to continue with our online community’s instance. In a manner comparable to this, we could establish an attribute (edge) for every connection that measures the degree of familiarity between the two individuals or the nature of the connection (e.g., family-related, professional, etc.). Each of the vertices and edges may in fact have a variety of qualities to take into account, therefore they are denoted by numerical vectors of attributes with the $v_{i}^{A}$ and $e_{i,j}^{A}$, correspondingly. Below stated Fig. 2 is a sample graph represents connections in the online community.

The method of maintaining or changing any value in an automated process or other piece of scientific machinery in accordance with predetermined circumstances without having the direct involvement of a human being is known as *automatic control*. Automatic controllers are devices that are assembled specifically for this use. The research and development of techniques for formulating regulations for managing systems that are able to be implemented by automated technologies. These techniques have traditionally been used primarily for technical procedures (see^1–5^). As a result, an airplane in motion is a system whose control principles guarantee that it stays on the necessary trajectories. The automated pilot, which consists of transducers (measuring instruments) and actuators, is used to implement the rules and regulations.

The concept of optimal control is concerned with addressing the issue of determining the control principle for an instance of a system that meets a specific optimality condition. A cost function the fact that depends on stipulate and control parameters is a component of a control issue. A collection of mathematical models characterizing the movements of the parameters under control in a way that minimizes the cost function is referred to as an *optimal control*. Numerous optimum control problems have been studied using it extensively in the literature (see^6–8^). In both the natural and applied sciences, the contractive mapping concept is one of the most effective methods for examining and/or solving existence results. The idea, which is additionally referred to as the fixed point problem, suggests that a recursive strategy provided by repeating representations under the mapping of any initial point in the metric space would always have a single fixed point that is able to be identified as the limit. The contractive mapping hypothesis is one that has been widely refined at every step of the course of history in many theoretical disciplines (see^9–14^), and currently holds significance for complex scientific fields, computation algorithms, and numerical models.

The objective of this paper is to study the automatic flight path generation of a control function and optimum control in the context of graphical notions and nonlinear graphical supra-contractive mappings.

## Primary details on graphical notions

Here, we provide a summary of a few fundamental concepts and practical findings that can be used as methods for further investigation. In addition, let ${\mathscr {M}}$ be a set that is not empty, and take $\triangle$ be the diagonal generated by ${\mathscr {M}}\times {\mathscr {M}}$. Assume that the directed graph ${\mathscr {G}}$ has zero parallel edges. The vertices of ${\mathscr {V}}({\mathscr {G}})$ has to correspond through ${\mathscr {M}}$, and ${\mathcal {E}}({\mathscr {G}})\sqsubseteq {\mathscr {M}}\times {\mathscr {M}}$ is required to contain all loops. This makes $({\mathscr {V}}({\mathscr {G}}),{\mathcal {E}}({\mathscr {G}}))$ is used to identify ${\mathscr {G}}$. The graph ${\mathscr {G}}^{-1}$ is referred to as a graph generated by switching to the reverse direction of its edges from ${\mathscr {G}}$.

In other words:

$$\begin{aligned} {\mathcal {E}}({\mathscr {G}}^{-1})=\{({\mathfrak {a}},\mathbbm {b})\in {\mathscr {M}}\times {\mathscr {M}}:(\mathbbm {b},{\mathfrak {a}})\in {\mathcal {E}}({\mathscr {G}})\}. \end{aligned}$$

It is less restrictive when considering $\widetilde{{\mathscr {G}}}$ and the collection of edges is symmetrical. With such a method, we produce the following:

$$\begin{aligned} {\mathcal {E}}(\widetilde{{\mathscr {G}}})={\mathcal {E}}({\mathscr {G}})\sqcup {\mathcal {E}}({\mathscr {G}}^{-1}). \end{aligned}$$

If ${\mathfrak {a}},\mathbbm {b}$ are vertices of ${\mathscr {G}}$, and subsequently a path in ${\mathscr {G}}$ is a sequence ${\mathfrak {a}}_{i=0}^{n}$ of $(n+1)$ vertices in such a way that ${\mathfrak {a}}_{0}={\mathfrak {a}}, \ {\mathfrak {a}}_{n}=\mathbbm {b}$ and the pair $({\mathfrak {a}}_{i-1},{\mathfrak {a}}_{i})$ should be in ${\mathcal {E}}({\mathscr {G}})$ where $i=1,2,3,\ldots n.$ In order for a graph ${\mathscr {G}}$ to be considered to be *connected*-a minimum of one path must connect each pair of its vertices. If there exists a minimum of one directed path connecting each vertex to each subsequent one in a directed graph-it has been hypothesized to be *strongly connected*. If the corresponding undirected graph of a directed graph ${\mathscr {G}}$ is connected nevertheless ${\mathscr {G}}$ fails to be strongly connected, the directed graph is said to be weakly connected. The set of all vertices and edges of ${\mathscr {A}}$ are subsets of ${\mathscr {G}}$; where ${\mathscr {A}}$ is a subgraph of graph ${\mathscr {G}}$. If a vertex is present in ${\mathscr {A}}$, it has a corresponding vertex in ${\mathscr {G}}$, and any edge that connects two vertices in ${\mathscr {A}}$ will also connect the corresponding vertices in ${\mathscr {G}}$. However, not all of the edges and not all of the vertices of ${\mathscr {G}}$ are required to exist in ${\mathscr {A}}$.

In order to generalize the metric space by utilizing graphical notions, Shukla et al.,^15^ initially put forward the idea of graphical metric space:

### Definition 1

Suppose that ${\mathscr {M}}$ be a set that is nonempty and is endowed with the graph ${\mathscr {G}}$. Let ${\mathscr {G}}_{m}:{\mathscr {M}}\times {\mathscr {M}}\rightarrow {\mathbb {R}}$ be a function gratifying the below mentioned assertions:

$$({\mathscr {G}}_{1})$$
$$(\forall \ {\mathfrak {a}},\mathbbm {b}\in {\mathscr {M}}) \ {\mathscr {G}}_{m}({\mathfrak {a}},\mathbbm {b})\ge 0;$$
$$({\mathscr {G}}_{2})$$
$$(\forall \ {\mathfrak {a}},\mathbbm {b}\in {\mathscr {M}}) \ {\mathscr {G}}_{m}({\mathfrak {a}},\mathbbm {b})=0 \ \text {iff} \ {\mathfrak {a}}=\mathbbm {b};$$
$$({\mathscr {G}}_{3})$$
$$(\forall \ {\mathfrak {a}},\mathbbm {b}\in {\mathscr {M}}) \ {\mathscr {G}}_{m}({\mathfrak {a}},\mathbbm {b})={\mathscr {G}}_{m}(\mathbbm {b},{\mathfrak {a}});$$
$$({\mathscr {G}}_{4})$$
$$({\mathfrak {a}}{\textbf{P}}\mathbbm {b})_{{\mathscr {G}}},\texttt {c}\in ({\mathfrak {a}}{\textbf{P}}\mathbbm {b})_{{\mathscr {G}}} \ \text {implies} \ {\mathscr {G}}_{m}({\mathfrak {a}},\mathbbm {b})\le {\mathscr {G}}_{m}({\mathfrak {a}},\texttt {c})+{\mathscr {G}}_{m}(\texttt {c}, \mathbbm {b}); $$ $$\text {where} {\mathfrak {a}},\mathbbm {b},\texttt {c} \ \text {are any three elements of} \ {\mathscr {M}}.$$

The pair $({\mathscr {M}},{{\mathscr {G}}_{m}})$ is referred to as a graphical metric space, whereas the mapping ${\mathscr {G}}_{m}$ is referred to as a graphical metric over ${\mathscr {M}}$.

Adopting various “*triangular inequalities*” with a provided set yields distinct graphical metric spaces:

$$({T_{1}}).$$
For some $$s\ge 1 \ \text {and for all} \ {\mathfrak {a}},\mathbbm {b},\texttt {c}\in {\mathscr {M}} \ \text {such that} \ $$ $$({\mathfrak {a}}{\textbf{P}}\mathbbm {b})_{{\mathscr {G}}},\texttt {c}\in ({\mathfrak {a}}{\textbf{P}}\mathbbm {b})_{{\mathscr {G}}} \ \text {which implies}$$ $$ {{\mathscr {G}}_{m}}({\mathfrak {a}},\mathbbm {b})\le s[{{\mathscr {G}}_{m}}({\mathfrak {a}},\texttt {c})+{{\mathscr {G}}_{m}}(\texttt {c}, \mathbbm {b})];$$.
$$({T_{2}}).$$
There exists a $$\theta \in {\mathbb {R}}^{+} \ \text {and for all} \ {\mathfrak {a}},\mathbbm {b},\texttt {c}\in {\mathscr {M}} \ \text {such that} \ ({\mathfrak {a}}{\textbf{P}}\mathbbm {b})_{{\mathscr {G}}},\texttt {c}\in ({\mathfrak {a}}{\textbf{P}}\mathbbm {b})_{{\mathscr {G}}} \ \text {which implies} $$ $$ {{\mathscr {G}}_{m}}({\mathfrak {a}},\mathbbm {b})\le {{\mathscr {G}}_{m}}({\mathfrak {a}},\texttt {c})+{{\mathscr {G}}_{m}}(\texttt {c}, \mathbbm {b})+\theta {{\mathscr {G}}_{m}}({\mathfrak {a}},\texttt {c}){{\mathscr {G}}_{m}}(\texttt {c}, \mathbbm {b})$$;
$$({T_{3}}).$$
There exists a function $\vartheta :{\mathscr {M}}\times {\mathscr {M}}\rightarrow [1,+\infty ) \ \text {and for all} \ {\mathfrak {a}},\mathbbm {b},\texttt {c}\in {\mathscr {M}} \ \text {such that} \ ({\mathfrak {a}}{\textbf{P}}\mathbbm {b})_{{\mathscr {G}}},\texttt {c}\in ({\mathfrak {a}}{\textbf{P}}\mathbbm {b})_{{\mathscr {G}}}$ $\text {which implies} \ {{\mathscr {G}}_{m}}({\mathfrak {a}},\mathbbm {b})\le {{\mathscr {G}}_{m}}({\mathfrak {a}},\texttt {c})+{{\mathscr {G}}_{m}}(\texttt {c}, \mathbbm {b})+\vartheta ({\mathfrak {a}},\mathbbm {b}) {{\mathscr {G}}_{m}}({\mathfrak {a}},\texttt {c}){{\mathscr {G}}_{m}}(\texttt {c}, \mathbbm {b})$.

$${\mathscr {G}}$$ is called,

(*i*): Graphical *b*-metric^16^ if we take $({T_{1}})$ instead of $({\mathscr {G}}_{4})$ in Definition 1.
(*ii*): Graphical suprametric if we take $({T_{2}})$ instead of $({\mathscr {G}}_{4})$ in Definition 1.
(*iii*): An extended graphical suprametric if we take $({T_{3}})$ instead of $({\mathscr {G}}_{4})$ in Definition 1.

The pair $({\mathscr {M}},{\mathscr {G}})$ is said to be a graphical *b*-metric/graphical suprametric/extended graphical suprametric space if ${\mathscr {G}}$ is a *b*-metric/graphical suprametric/extended graphical suprametric on ${\mathscr {M}}$.

As a unification of graphical metric space^15^, suprametric space^17^ and an extended suprametric space^18^, we first introduce the topics of graphical suprametric space and an extended graphical suprametric space as above.

Considering a relation ${\textbf{P}}$ stated that over ${\mathscr {M}},({\mathfrak {a}}{\textbf{P}}\mathbbm {b})_{{\mathscr {G}}}$ and $[{\mathfrak {a}}]_{{\mathscr {G}}}^{\ell }$ are able to be characterized as follows:

1. $({\mathfrak {a}}{\textbf{P}}\mathbbm {b})_{{\mathscr {G}}}\Leftrightarrow$ there exist a path in the direction from ${\mathfrak {a}}$ to $\mathbbm {b}$ in ${\mathscr {G}}$ and $\texttt {c}\in ({\mathfrak {a}}{\textbf{P}}\mathbbm {b})_{{\mathscr {G}}}$ if $\texttt {c}$ is having a path in the direction from ${\mathfrak {a}}$ to $\mathbbm {b}$ in ${\mathscr {G}}$;
2. $[{\mathfrak {a}}]_{{\mathscr {G}}}^{\ell }=\{\mathbbm {b}\in {\mathscr {M}}:$ there exists a directed path from ${\mathfrak {a}}$ to $\mathbbm {b}$ of length $\ell \ \}$, for more info, refer^16^.

Moreover, a sequence $\{{\mathfrak {a}}_{n}\}\in {\mathscr {M}}$ is called ${\mathscr {G}}$-termwise connected whenever $({\mathfrak {a}}_{n}{\textbf{P}}{\mathfrak {a}}_{n+1}){\mathscr {G}}$ where *n* should be in ${\mathbb {N}}$.

### Remark 2

An extended suprametric space $({\mathscr {M}},{\mathscr {G}})$ is an extended graphical suprametric space having graph ${\mathscr {G}}$, whenever ${\mathscr {V}}({\mathscr {G}})={\mathscr {M}}$ and ${\mathcal {E}}({\mathscr {G}})={\mathscr {M}}\times {\mathscr {M}}.$

It is worth noticing that we introduced an extended graphical suprametric space as a generalization of an extended suprametric space^18^.

### Example 3

Take ${\mathscr {M}}=\{0,1,2,3\}$. Define the function ${{\mathscr {G}}_{m}}:{\mathscr {M}}\times {\mathscr {M}}\rightarrow {\mathbb {R}}^{+}$ as ${{\mathscr {G}}_{m}}(1,1)={{\mathscr {G}}_{m}}(2,2)={{\mathscr {G}}_{m}}(3,3)={{\mathscr {G}}_{m}}(0,0)=0$ and ${{\mathscr {G}}_{m}}({\mathfrak {a}},\mathbbm {b})={{\mathscr {G}}_{m}}(\mathbbm {b},{\mathfrak {a}})$ for all ${\mathfrak {a}},\mathbbm {b}\in {\mathscr {M}}$,

**Table:** 

${{\mathscr {G}}_{m}}$	0	1	2	3
0	0	0.125	0.111	0.1
1	0.125	0	0.090	0.083
2	0.111	0.090	0	0.142
3	0.001	0.083	0.142	0

**Figure 3:**
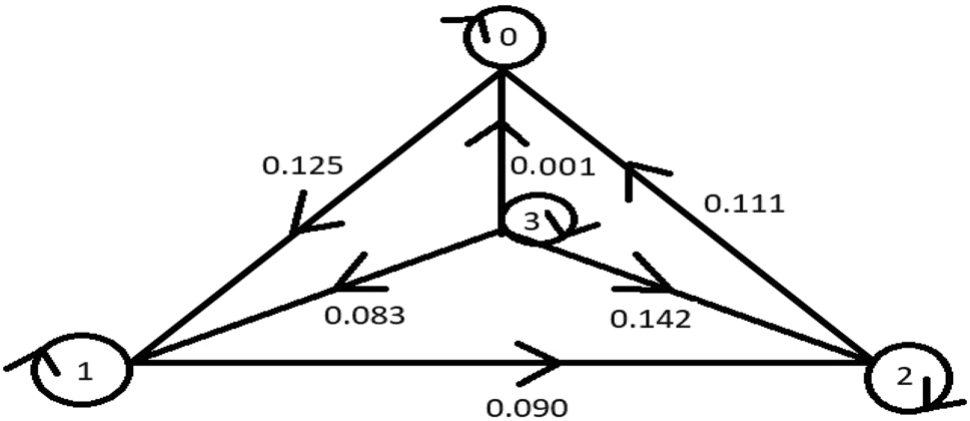
Plot for extended graphical suprametric space.

Define $\vartheta :{\mathscr {M}}\times {\mathscr {M}}\rightarrow [1,\infty )$ by $\vartheta ({\mathfrak {a}},\mathbbm {b})=e ^{{\mathfrak {a}}+\mathbbm {b}}$. Analyze the graph ${\mathscr {G}}$ with ${\mathscr {M}}={\mathscr {V}}({\mathscr {G}})$ as the collection of vertices and the edge set provided below:

$$\begin{aligned} {\mathcal {E}}({\mathscr {G}})=\triangle \sqcup \{(0,1),(0,2), (0,3),(1,2),(1,3),(2,3)\}, \end{aligned}$$

then $({\mathscr {M}},{\mathscr {G}})$ is an extended graphical suprametric space with $\vartheta ({\mathfrak {a}},\mathbbm {b})=e ^{{\mathfrak {a}}+\mathbbm {b}}$, confining the graph ${\mathscr {G}}$ as explained in below Fig. 3.

### Remark 4

It is important to mention that graphical suprametric space can be an extended graphical suprametric space by taking $\vartheta ({\mathfrak {a}},\mathbbm {b})=\theta$ (where $\theta \ge 1$) a constant function (where the mapping $\vartheta$ should map to a value greater than or equal to 1). But the converse may not be true. The following example supports our argument.

### Example 5

**Figure 4:**
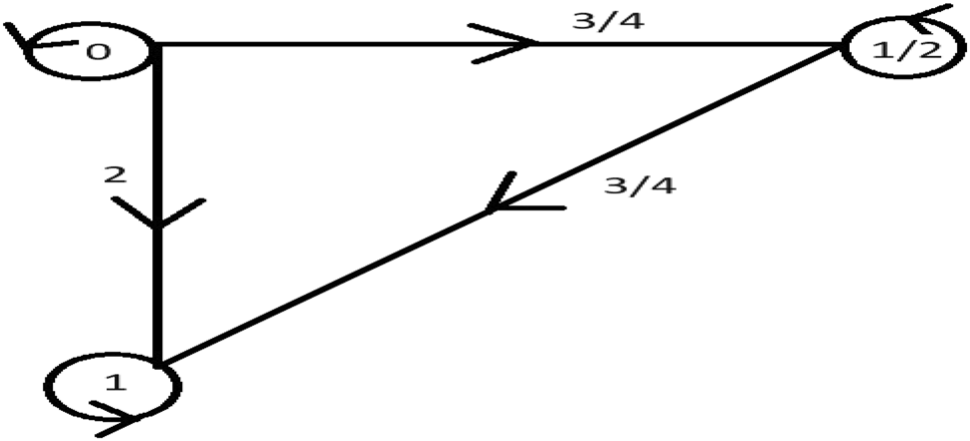
Plot for extended suprametric space.

Take ${\mathscr {M}}=\{0,\frac{1}{2},1\}$ and $\mu$ be a graph metric on ${\mathscr {M}}$ defined as $\mu ({\mathfrak {a}},\mathbbm {b})=|{\mathfrak {a}}-\mathbbm {b}|$ for all ${\mathfrak {a}},\mathbbm {b}\in {\mathscr {M}}$. Define ${{\mathscr {G}}_{m}}:{\mathscr {M}}\times {\mathscr {M}}\rightarrow {\mathbb {R}}^{+}$ as ${{\mathscr {G}}_{m}}^{\eta }({\mathfrak {a}},\mathbbm {b})=\mu ({\mathfrak {a}}, \mathbbm {b})(\mu ({\mathfrak {a}},\mathbbm {b})+\eta )$ for any real number $\eta .$ Define $\vartheta :{\mathscr {M}}\times {\mathscr {M}}\rightarrow [1,\infty )$ by $\vartheta ({\mathfrak {a}},\mathbbm {b})=e ^{{\mathfrak {a}}+\mathbbm {b}}$. Let ${\mathscr {M}}$ be endowed with a graph ${\mathscr {G}}$ with ${\mathscr {M}}={\mathscr {V}}({\mathscr {G}})$ as the collection of vertices and the edge set provided below:

$$\begin{aligned} {\mathcal {E}}({\mathscr {G}})=\triangle \sqcup \{(0,\frac{1}{2}),(0,1),(\frac{1}{2},1)\}, \end{aligned}$$

then $({\mathscr {M}},{\mathscr {G}})$ is an extended suprametric space with $\vartheta ({\mathfrak {a}},\mathbbm {b})=e ^{{\mathfrak {a}}+\mathbbm {b}}$ and $\eta =1$, confining the graph ${\mathscr {G}}$ as explained in Fig. 4. But it is not an graphical suprametric space with $\theta =\frac{1}{3}$

as $${{\mathscr {G}}_{m}}(0,1)>{{\mathscr {G}}_{m}}(0,\frac{1}{2}) +{{\mathscr {G}}_{m}}(\frac{1}{2},1)+\frac{1}{3}{{\mathscr {G}}_{m}} (0,\frac{1}{2}){{\mathscr {G}}_{m}}(\frac{1}{2},1)$$.

**Figure 5:**
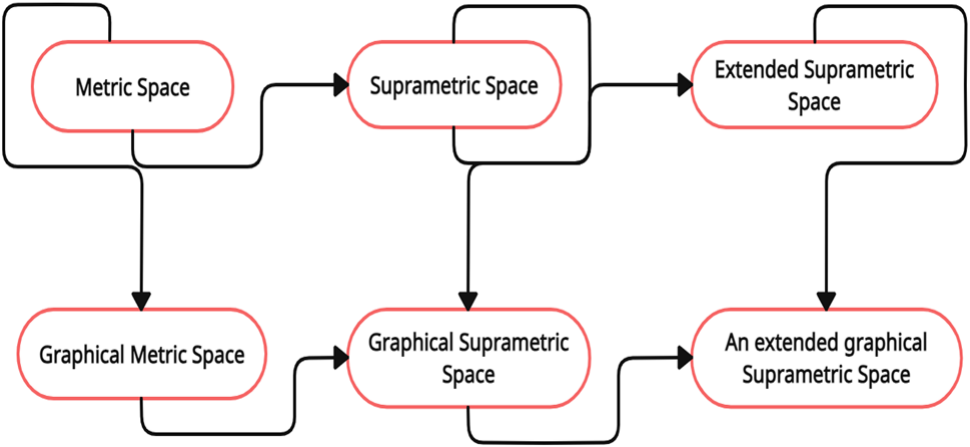
Plot for graphical framework of suprametric and/or extended-suprametric.

Using renowned literature^19–22^ and the work done in this article, we develop subsequent Fig. 5 to better understand the graphical framework of suprametric and/or extended-suprametric spaces. Moreover, in below Fig. 5, inclusion is represented by an arrow. But however, reverse inclusion is not true.

We define open and closed balls as follows in order to investigate the topological structure of an extended graphical suprametric space.

### Definition 6

Consider a extended graphical suprametric space $({\mathscr {M}},{{\mathscr {G}}_{m}})$. Take ${\mathfrak {a}}\in {\mathscr {M}}$ and $\varepsilon >0$. Then ${{\mathscr {G}}_{m}}$-open ball having center ${\mathfrak {a}}_{0}$ and radius $\varepsilon$ is

$$\begin{aligned} {\mathscr {B}}_{{{\mathscr {G}}_{m}}}({\mathfrak {a}},\varepsilon )=\{\mathbbm {b}\in {\mathscr {M}}/({\mathfrak {a}}{\textbf{P}}\mathbbm {b})_{{\mathscr {G}}}, \ {{\mathscr {G}}_{m}}({\mathfrak {a}},\mathbbm {b})<\varepsilon \}. \end{aligned}$$

Similarly, ${{\mathscr {G}}_{m}}$-closed ball having center ${\mathfrak {a}}_{0}$ and radius $\varepsilon$ is

$$\begin{aligned} {\mathscr {B}}_{{{\mathscr {G}}_{m}}}({\mathfrak {a}},\varepsilon )=\{\mathbbm {b}\in {\mathscr {M}}/({\mathfrak {a}}{\textbf{P}}\mathbbm {b})_{{\mathscr {G}}}, \ {{\mathscr {G}}_{m}}({\mathfrak {a}},\mathbbm {b})\le \varepsilon \}. \end{aligned}$$

As $\triangle \sqsubseteq {\mathcal {E}}({\mathscr {G}}),$ which implies that ${\mathfrak {a}}\in {\mathscr {B}}_{{{\mathscr {G}}_{m}}}({\mathfrak {a}},\varepsilon )$ and so ${\mathscr {B}}_{{{\mathscr {G}}_{m}}}({\mathfrak {a}},\varepsilon )\ne \emptyset$ for all ${\mathfrak {a}}\in {\mathscr {M}}$ and $\varepsilon >0$. Moreover, the set ${\mathcal {B}}=\{{\mathscr {B}}_{{{\mathscr {G}}_{m}}}({\mathfrak {a}},\varepsilon )/{\mathfrak {a}}\in {\mathscr {M}} , \varepsilon >0\}$ builds a neighbourhood system.

### Definition 7

Let $({\mathscr {M}},{{\mathscr {G}}_{m}})$ be an extended graphical suprametric space. ${\mathscr {A}}\sqsubseteq {\mathscr {M}}$ is said to be *open* if for all ${\mathfrak {a}}\in {\mathscr {A}}$ there is a $\varepsilon >0$ so that ${\mathscr {B}}_{{{\mathscr {G}}_{m}}}({\mathfrak {a}},\varepsilon )\subset {\mathscr {A}}$. Generally ${\mathscr {A}}\sqsubseteq {\mathscr {M}}$ is known as closed whenever its complement is open.

### Proposition 8

Suppose $({\mathscr {M}},{{\mathscr {G}}_{m}})$ be an extended graphical suprametric space. Take ${\mathfrak {a}}\in {\mathscr {M}}$ and $\varepsilon >0$ with $\vartheta ({\mathfrak {a}},\mathbbm {b})=\kappa \in [0,\infty )$, for all ${\mathfrak {a}},\mathbbm {b}\in {\mathscr {M}}$. If $\mathbbm {b}\in {\mathscr {B}}_{{{\mathscr {G}}_{m}}}({\mathfrak {a}},\varepsilon )$ then there exist $\wp >0$ such that ${\mathscr {B}}_{{{\mathscr {G}}_{m}}}(\mathbbm {b},\wp )\sqsubseteq {\mathscr {B}}_{{{\mathscr {G}}_{m}}}({\mathfrak {a}},\varepsilon )$.

Proof. Let $\mathbbm {b}\in {\mathscr {B}}_{{{\mathscr {G}}_{m}}}({\mathfrak {a}},\varepsilon )$ and if $\mathbbm {b}={\mathfrak {a}}$ then we choose $\wp =\varepsilon$.

Suppose that $\wp \ne \varepsilon$, then we get ${{\mathscr {G}}_{m}}({\mathfrak {a}},\mathbbm {b})\ne 0$. We choose $\wp =\frac{\varepsilon -{{\mathscr {G}}_{m}}({\mathfrak {a}}, \mathbbm {b})}{1+\kappa {{\mathscr {G}}_{m}}({\mathfrak {a}},\mathbbm {b})}>0$ and let $\texttt {c}\in {\mathscr {B}}_{{{\mathscr {G}}_{m}}}(\mathbbm {b},\wp )$. Therefore, in accordance with the stated hypothesis, we have $({\mathfrak {a}}{\textbf{P}}\mathbbm {b})_{{\mathscr {G}}}$ and $(\mathbbm {b}{\textbf{P}}{} \texttt {c})_{{\mathscr {G}}}$ and so $({\mathfrak {a}}{\textbf{P}}{} \texttt {c})_{{\mathscr {G}}}$. Consequently, the triangle inequality $({T_{3}})$ demonstrates that

$$\begin{aligned} {{\mathscr {G}}_{m}}(\texttt {c},{\mathfrak {a}})&\le {{\mathscr {G}}_{m}}(\texttt {c},\mathbbm {b})+{{\mathscr {G}}_{m}} (\mathbbm {b},{\mathfrak {a}})+\vartheta (\texttt {c},{\mathfrak {a}}) {{\mathscr {G}}_{m}}(\texttt {c},\mathbbm {b}){{\mathscr {G}}_{m}}(\mathbbm {b},{\mathfrak {a}})\\&<\wp +{{\mathscr {G}}_{m}}(\mathbbm {b},{\mathfrak {a}})+\kappa \wp {{\mathscr {G}}_{m}}(\mathbbm {b},{\mathfrak {a}})\\&=\wp (1+\kappa {{\mathscr {G}}_{m}}(\mathbbm {b},{\mathfrak {a}})) +{{\mathscr {G}}_{m}}(\mathbbm {b},{\mathfrak {a}})\\&=\frac{\varepsilon -{{\mathscr {G}}_{m}}(\mathbbm {b},{\mathfrak {a}})}{1+\kappa {{\mathscr {G}}_{m}}(\mathbbm {b},{\mathfrak {a}})} (1+\kappa {{\mathscr {G}}_{m}}(\mathbbm {b},{\mathfrak {a}})) +{{\mathscr {G}}_{m}}(\mathbbm {b},{\mathfrak {a}})\\&=\varepsilon . \end{aligned}$$

Hence ${{\mathscr {G}}_{m}}(\texttt {c},{\mathfrak {a}})<\varepsilon$. Which yields ${\mathscr {B}}_{{{\mathscr {G}}_{m}}}(\mathbbm {b},\wp )\sqsubseteq {\mathscr {B}}_{{{\mathscr {G}}_{m}}}({\mathfrak {a}},\varepsilon )$. This illustrates that each open ball in ${\mathscr {M}}$ is an open set.

### Proposition 9

Let $({\mathscr {M}},{{\mathscr {G}}_{m}})$ be an extended graphical suprametric space. Let $\tau$ be the family of all open subsets of ${\mathscr {M}}$. Then $\tau$ is a topology on ${\mathscr {M}}$.

### Definition 10

Let $({\mathscr {M}},{{\mathscr {G}}_{m}})$ be an extended graphical suprametric space.

1. A sequence $\{{\mathfrak {a}}_{n}\}$ converges to some ${\mathfrak {a}}$ in ${\mathscr {M}}$. If for all positive $\varepsilon$, there is some positive $N_{\varepsilon }$ such that ${{\mathscr {G}}_{m}}({\mathfrak {a}}_{n},{\mathfrak {a}})<\varepsilon$ for each $n\ge N_{\varepsilon }$. It can be written as, $\lim _{n\rightarrow \infty }{\mathfrak {a}}_{n}={\mathfrak {a}}$.
2. The sequence $\{{\mathfrak {a}}_{n}\}$ in an extended graphical suprametric space $({\mathscr {M}},{{\mathscr {G}}_{m}})$ is said to be a Cauchy sequence if ${{\mathscr {G}}_{m}}({\mathfrak {a}}_{n},{\mathfrak {a}}_{m})\rightarrow 0$ as $n,m\rightarrow \infty$.
3. A extended graphical suprametric space $({\mathscr {M}},{{\mathscr {G}}_{m}})$ is said to be complete if every Cauchy sequence is convergent in ${\mathscr {M}}$ with respect to graph ${\mathscr {G}}$.

### Example 11

Let ${\mathfrak {a}}_{n}=\frac{1}{2^{n}}$ for $n\in {\mathbb {N}}$ be a sequence in an extended graphical suprametric space $({\mathscr {M}},{{\mathscr {G}}_{m}})$.

Let $\varepsilon =0.1$ and ${\mathfrak {a}}=0$ then ${{\mathscr {G}}_{m}}({\mathfrak {a}}_{n},0)=|\frac{1}{2^{n}}-0|=\frac{1}{2^{n}}<\varepsilon$.

i.e., $$2^{n}>\frac{1}{\varepsilon }\Rightarrow \ n>\frac{\log (\frac{1}{\varepsilon })}{\log (2)}\Rightarrow n>3.32$$.

Thus for $\varepsilon =0.1>0$ there exists $4=m\in {\mathbb {N}}$ such that ${{\mathscr {G}}_{m}}({\mathfrak {a}}_{n},{\mathfrak {a}})<\varepsilon$ for all $n\ge 4$. Thus, the sequence ${\mathfrak {a}}_{n}=\frac{1}{2^{n}}$ converges to “0” in an extended graphical suprametric space $({\mathscr {M}},{{\mathscr {G}}_{m}})$.

## Graphical supra-contractive mappings

As a generalization of the idea of graphical contraction in distance spaces, we introduce a subsequent definition at the outset of this section.

### Definition 12

Let $({\mathscr {M}},{{\mathscr {G}}_{m}})$ be an extended graphical suprametric space. Take ${\mathscr {U}}:{\mathscr {M}}\rightarrow {\mathscr {M}}$ is a mapping on ${\mathscr {M}}$. Moreover, let ${\mathscr {G}}^{\star }$ be a subgraph of ${\mathscr {G}}$ so that $\triangle$ is a subset of ${\mathcal {E}}({\mathscr {G}}^{\star })$. ${\mathscr {U}}$ is a supra-graphical contraction on extended graphical suprametric space ${\mathscr {M}}$, if and only if the following conditions are met:

$${\mathcal {C}}_{1}$$
. ${\mathscr {U}}$ having edges of ${\mathscr {G}}^{\star }$, the same for each $({\mathfrak {a}},\mathbbm {b})\in {\mathcal {E}}({\mathscr {G}}^{\star })$, which implies $({\mathscr {U}}{\mathfrak {a}},{\mathscr {U}}\mathbbm {b})\in {\mathcal {E}}({\mathscr {G}}^{\star })$;
$${\mathcal {C}}_{2}$$
. there exists $\gamma \in [0,1)$ and $\vartheta ({\mathfrak {a}},\mathbbm {b})\in [1,\infty )$ for all ${\mathfrak {a}},\mathbbm {b}\in {\mathscr {M}}$ with $({\mathfrak {a}},\mathbbm {b})\in {\mathcal {E}}({\mathscr {G}}^{\star })$ such that
  1 $$\begin{aligned} {{\mathscr {G}}_{m}}({\mathscr {U}}{\mathfrak {a}},{\mathscr {U}}\mathbbm {b})\le \gamma {{\mathscr {G}}_{m}}({\mathfrak {a}},\mathbbm {b}). \end{aligned}$$

It is worth noting that ${\mathscr {G}}^{\star }$ can be regarded is a weighted graph as it refers to every edge by the distance among their corresponding vertices. The ${\mathscr {U}}$-Picard iterative sequence $\{{\mathfrak {a}}_{n}\}$ is defined as ${\mathfrak {a}}_{n}={\mathscr {U}}{\mathfrak {a}}_{n-1}$ for all $n\in {\mathbb {N}}$ with initial value ${\mathfrak {a}}_{0}\in {\mathscr {M}}$. For the sake of further investigation, suppose that $\triangle \sqsubseteq {\mathcal {E}}({\mathscr {G}}^{\star })$ and that ${\mathscr {G}}^{\star }$ is a subgraph of ${\mathscr {G}}$. We’ll also make use of the subsequent result.

${\mathscr {G}}^{\star }$ is called to be gratify the property $({\textbf{P}}^{\star })$, if a ${\mathscr {G}}^{\star }$-termwise connected ${\mathscr {U}}$-Picard sequence $\{{\mathfrak {a}}_{n}\}$ converges in ${\mathscr {M}}$ which yields $\texttt {c}\in {\mathscr {M}}$ of $\{{\mathfrak {a}}_{n}\}$ and $n_{0}\in {\mathbb {N}}$ such that $({\mathfrak {a}}_{n},\texttt {c})\in {\mathcal {E}}({\mathscr {G}}^{\star })$ or $(\texttt {c},{\mathfrak {a}}_{n})\in {\mathcal {E}}({\mathscr {G}}^{\star })$ for all $n>n_{0}$.

### Theorem 13

Assume that $({\mathscr {M}},{{\mathscr {G}}_{m}})$ be a complete extended graphical suprametric space and ${\mathscr {U}}:{\mathscr {M}}\rightarrow {\mathscr {M}}$ be a supra-graphical contraction on ${\mathscr {M}}$. Consider that the subsequent assertions are true:

$${\mathscr {G}}_{1}.$$
There exists ${\mathfrak {a}}_{0}\in {\mathscr {M}}$ such that ${\mathscr {U}}{\mathfrak {a}}_{0}\in [{\mathfrak {a}}_{0}]_{{\mathscr {G}}^{\star }}^{\ell }$, where $\ell$ should be in ${\mathbb {N}}.$
$${\mathscr {G}}_{2}.$$
The graph ${\mathscr {G}}^{\star }$ gratifies the property $({\textbf{P}}^{\star })$, and ${\mathfrak {a}}^{\star }\in {\mathscr {M}}$ in such a way that the ${\mathscr {U}}$-Picard sequence $\{{\mathfrak {a}}_{n}\}$ with ${\mathfrak {a}}_{0}\in {\mathscr {M}}$ is ${\mathscr {G}}^{\star }$-termwise connected. Furthermore, it is converges to ${\mathfrak {a}}^{\star }$ and ${\mathscr {U}}{\mathfrak {a}}^{\star }$.
$${\mathscr {G}}_{3}.$$
The quadruple $({\mathscr {M}},{{\mathscr {G}}_{m}},{\mathscr {G}}^{\star },{\mathscr {U}})$ satisfies the property $({\textbf{P}})$, i.e., if ${\mathscr {G}}^{\star }$-termwise connected ${\mathscr {U}}$-Picard sequence $\{{\mathfrak {a}}_{n}\}$ having limits ${\mathfrak {a}}^{\star },\mathbbm {b}^{\star }$, in which ${\mathfrak {a}}^{\star }\in {\mathscr {M}}$ and $\mathbbm {b}^{\star }\in {\mathscr {U}}({\mathscr {M}})$, which implies ${\mathfrak {a}}^{\star }=\mathbbm {b}^{\star }$.

As a result, ${\mathscr {U}}$ has a fixed point.

Proof. Starting from ${\mathfrak {a}}_{0}\in {\mathscr {M}}$ such that ${\mathscr {U}}{\mathfrak {a}}_{0}\in [{\mathfrak {a}}_{0}]_{{\mathscr {G}}^{\star }}^{\ell }$ and utilizing the sequence $\{{\mathfrak {a}}_{n}\}$ with initial value ${\mathfrak {a}}_{0}$, then there is a path $\{\mathbbm {b}_{i}\}_{i=0}^{\ell }$ in such a way that ${\mathfrak {a}}_{0}=\mathbbm {b}_{0}$, ${\mathscr {U}}{\mathfrak {a}}_{0}=\mathbbm {b}_{\ell }$ and $(\mathbbm {b}_{i-1},\mathbbm {b}_{i})\in {\mathcal {E}}({\mathscr {G}}^{\star })$ where $i=1,2,3\ldots \ell$.

As ${\mathscr {U}}$ holds edges in ${\mathscr {G}}^{\star }$, from the assertion of ${\mathcal {C}}_{1}$, we know that $({\mathscr {U}}\mathbbm {b}_{i-1},{\mathscr {U}}\mathbbm {b}_{i})\in {\mathcal {E}}({\mathscr {G}}^{\star })$ for $i=1,2,3\ldots \ell$. Thus $\{{\mathscr {U}}\mathbbm {b}_{i}\}_{i=0}^{\ell }$ is having a length $\ell$ from ${\mathscr {U}}\mathbbm {b}_{0}={\mathscr {U}}{\mathfrak {a}}_{0}={\mathfrak {a}}_{1}$ to ${\mathscr {U}}\mathbbm {b}_{1}={\mathscr {U}}^{2}{\mathfrak {a}}_{0}={\mathfrak {a}}_{2}$, which gives ${\mathfrak {a}}_{2}\in [{\mathfrak {a}}_{1}]_{{\mathscr {G}}^{\star }}^{\ell }$. By repeating this process we get $\{{\mathscr {U}}^{n}\mathbbm {b}_{i}\}_{i=0}^{\ell }$ is a path from ${\mathscr {U}}_{n}\mathbbm {b}_{0}={\mathscr {U}}^{n}{\mathfrak {a}}_{0}={\mathfrak {a}}_{n}$ to ${\mathscr {U}}^{n}\mathbbm {b}_{\ell }={\mathscr {U}}^{n}{\mathscr {U}}{\mathfrak {a}}_{0}={\mathfrak {a}}_{n+1}$ of length $\ell$, which yields ${\mathfrak {a}}_{n+1}\in [{\mathfrak {a}}_{n}]_{{\mathscr {G}}^{\star }}^{\ell }$, where *n* should be in ${\mathbb {N}}$.

As a result, $\{{\mathfrak {a}}_{n}\}$ becomes ${\mathscr {G}}^{\star }$-termwise connected sequence. As $({\mathscr {U}}^{n}\mathbbm {b}_{i-1},{\mathscr {U}}^{n}\mathbbm {b}_{i})\in {\mathcal {E}}({\mathscr {G}}^{\star })$ and by utilizing the supra-graphical contraction, we get,

2 $$\begin{aligned} {{\mathscr {G}}_{m}}({\mathscr {U}}^{n}\mathbbm {b}_{i-1},{\mathscr {U}}^{n}\mathbbm {b}_{i})\le \gamma {{\mathscr {G}}_{m}}({\mathscr {U}}^{n-1}\mathbbm {b}_{i-1},{\mathscr {U}}^{n-1}\mathbbm {b}_{i}). \end{aligned}$$

By continuing in the same process, we get,

$$\begin{aligned} {{\mathscr {G}}_{m}}({\mathscr {U}}^{n}\mathbbm {b}_{i-1},{\mathscr {U}}^{n}\mathbbm {b}_{i})\le \gamma ^{n} {{\mathscr {G}}_{m}}(\mathbbm {b}_{i-1},\mathbbm {b}_{i}). \end{aligned}$$

By the use of triangle inequality $({T_{3}})$, which yields that,

$$\begin{aligned} {{\mathscr {G}}_{m}}({\mathfrak {a}}_{n},{\mathfrak {a}}_{n+1})&={{\mathscr {G}}_{m}}({\mathscr {U}}^{n}{\mathfrak {a}}_{0},{\mathscr {U}}^{n+1}{\mathfrak {a}}_{0})\\&={{\mathscr {G}}_{m}}({\mathscr {U}}^{n}\mathbbm {b}_{0},{\mathscr {U}}^{n}\mathbbm {b}_{\ell })\\&\le {{\mathscr {G}}_{m}}({\mathscr {U}}^{n}\mathbbm {b}_{0},{\mathscr {U}}^{n}\mathbbm {b}_{1}) +{{\mathscr {G}}_{m}}({\mathscr {U}}^{n}\mathbbm {b}_{1},{\mathscr {U}}^{n}\mathbbm {b}_{\ell })\\&\ \ \ \ \ \ \ \ +\vartheta ({\mathscr {U}}^{n}\mathbbm {b}_{0},{\mathscr {U}}^{n}\mathbbm {b}_{\ell }) {{\mathscr {G}}_{m}}({\mathscr {U}}^{n}\mathbbm {b}_{0},{\mathscr {U}}^{n}\mathbbm {b}_{1}) {{\mathscr {G}}_{m}}({\mathscr {U}}^{n}\mathbbm {b}_{1},{\mathscr {U}}^{n}\mathbbm {b}_{\ell })\\&\le {{\mathscr {G}}_{m}}({\mathscr {U}}^{n}\mathbbm {b}_{0},{\mathscr {U}}^{n}\mathbbm {b}_{1}) +{{\mathscr {G}}_{m}}({\mathscr {U}}^{n}\mathbbm {b}_{1},{\mathscr {U}}^{n}\mathbbm {b}_{2}) +{{\mathscr {G}}_{m}}({\mathscr {U}}^{n}\mathbbm {b}_{2},{\mathscr {U}}^{n}\mathbbm {b}_{\ell })\\&\ \ \ \ \ \ \ \ +\vartheta ({\mathscr {U}}^{n}\mathbbm {b}_{1},{\mathscr {U}}^{n}\mathbbm {b}_{\ell }) {{\mathscr {G}}_{m}}({\mathscr {U}}^{n}\mathbbm {b}_{1},{\mathscr {U}}^{n}\mathbbm {b}_{2}) {{\mathscr {G}}_{m}}({\mathscr {U}}^{n}\mathbbm {b}_{2},{\mathscr {U}}^{n}\mathbbm {b}_{\ell })\\&\ \ \ \ \ \ \ \ +\vartheta ({\mathscr {U}}^{n}\mathbbm {b}_{0},{\mathscr {U}}^{n}\mathbbm {b}_{\ell }) {{\mathscr {G}}_{m}}({\mathscr {U}}^{n}\mathbbm {b}_{0},{\mathscr {U}}^{n}\mathbbm {b}_{1}) {{\mathscr {G}}_{m}}({\mathscr {U}}^{n}\mathbbm {b}_{1},{\mathscr {U}}^{n}\mathbbm {b}_{\ell }).\\ \end{aligned}$$

By repeating similar process, we get,

$$\begin{aligned} {{\mathscr {G}}_{m}}({\mathfrak {a}}_{n},{\mathfrak {a}}_{n+1})&\le {{\mathscr {G}}_{m}}({\mathscr {U}}^{n}\mathbbm {b}_{0},{\mathscr {U}}^{n}\mathbbm {b}_{1}) +{{\mathscr {G}}_{m}}({\mathscr {U}}^{n}\mathbbm {b}_{1},{\mathscr {U}}^{n}\mathbbm {b}_{2})+\cdots +{{\mathscr {G}}_{m}}({\mathscr {U}}^{n}\mathbbm {b}_{\ell -1},{\mathscr {U}}^{n}\mathbbm {b}_{\ell })\\&\ \ \ \ \ +\vartheta ({\mathscr {U}}^{n}\mathbbm {b}_{0},{\mathscr {U}}^{n}\mathbbm {b}_{\ell }) {{\mathscr {G}}_{m}}({\mathscr {U}}^{n}\mathbbm {b}_{0},{\mathscr {U}}^{n} \mathbbm {b}_{1}){{\mathscr {G}}_{m}}({\mathscr {U}}^{n}\mathbbm {b}_{1},{\mathscr {U}}^{n}\mathbbm {b}_{\ell })\\&\ \ \ \ \ +\vartheta ({\mathscr {U}}^{n}\mathbbm {b}_{1},{\mathscr {U}}^{n}\mathbbm {b}_{\ell }) {{\mathscr {G}}_{m}}({\mathscr {U}}^{n}\mathbbm {b}_{1},{\mathscr {U}}^{n} \mathbbm {b}_{2}){{\mathscr {G}}_{m}}({\mathscr {U}}^{n}\mathbbm {b}_{2},{\mathscr {U}}^{n}\mathbbm {b}_{\ell })\\&\ \ \ \ \ +\cdots +\vartheta ({\mathscr {U}}^{n}\mathbbm {b}_{\ell -2},{\mathscr {U}}^{n}\mathbbm {b}_{\ell }) {{\mathscr {G}}_{m}}({\mathscr {U}}^{n}\mathbbm {b}_{\ell -2},{\mathscr {U}}^{n} \mathbbm {b}_{\ell -1}){{\mathscr {G}}_{m}}({\mathscr {U}}^{n}\mathbbm {b}_{\ell -1} ,{\mathscr {U}}^{n}\mathbbm {b}_{\ell })\\&\le \sum _{i=1}^{\ell }{{\mathscr {G}}_{m}}({\mathscr {U}}^{n}\mathbbm {b}_{i-1}, {\mathscr {U}}^{n}\mathbbm {b}_{i})+\sum _{i=1}^{\ell -1}\vartheta ({\mathscr {U}}^{n} \mathbbm {b}_{i-1},{\mathscr {U}}^{n}\mathbbm {b}_{\ell }){{\mathscr {G}}_{m}} ({\mathscr {U}}^{n}\mathbbm {b}_{i-1},{\mathscr {U}}^{n}\mathbbm {b}_{i}){{\mathscr {G}}_{m}} ({\mathscr {U}}^{n}\mathbbm {b}_{i},{\mathscr {U}}^{n}\mathbbm {b}_{\ell })\\&\le \sum _{i=1}^{\ell }\gamma ^{n}{{\mathscr {G}}_{m}}(\mathbbm {b}_{i-1}, \mathbbm {b}_{i})+\sum _{i=1}^{\ell -1}\vartheta ({\mathscr {U}}^{n}\mathbbm {b}_{i-1}, {\mathscr {U}}^{n}\mathbbm {b}_{\ell })\gamma ^{n}{{\mathscr {G}}_{m}} (\mathbbm {b}_{i-1},\mathbbm {b}_{i}){{\mathscr {G}}_{m}}(\mathbbm {b}_{i},\mathbbm {b}_{\ell })\\&=\gamma ^{n}\bigg [ \sum _{i=1}^{\ell }{{\mathscr {G}}_{m}}(\mathbbm {b}_{i-1},\mathbbm {b}_{i}) +\sum _{i=1}^{\ell -1}\vartheta ({\mathscr {U}}^{n}\mathbbm {b}_{i-1},{\mathscr {U}}^{n}\mathbbm {b}_{\ell }) {{\mathscr {G}}_{m}}(\mathbbm {b}_{i-1},\mathbbm {b}_{i}){{\mathscr {G}}_{m}} (\mathbbm {b}_{i},\mathbbm {b}_{\ell })\bigg ].\\ \end{aligned}$$

Considering the insight that ${\mathscr {G}}^{\star }$-termwise connected sequence, we obtain:

$$\begin{aligned} {{\mathscr {G}}_{m}}({\mathfrak {a}}_{n},{\mathfrak {a}}_{m})&\le {{\mathscr {G}}_{m}}({\mathfrak {a}}_{n},{\mathfrak {a}}_{n+1})+{{\mathscr {G}}_{m}} ({\mathfrak {a}}_{n+1},{\mathfrak {a}}_{m})\\&\ \ \ \ \ \ +\vartheta ({\mathfrak {a}}_{n},{\mathfrak {a}}_{m}) {{\mathscr {G}}_{m}}({\mathfrak {a}}_{n},{\mathfrak {a}}_{n+1})+{{\mathscr {G}}_{m}} ({\mathfrak {a}}_{n+1},{\mathfrak {a}}_{m})\\&\le \gamma ^{n}\bigg [ \sum _{i=1}^{\ell }{{\mathscr {G}}_{m}}(\mathbbm {b}_{i-1},\mathbbm {b}_{i}) +\sum _{i=1}^{\ell -1}\vartheta ({\mathscr {U}}^{n}\mathbbm {b}_{i-1},{\mathscr {U}}^{n}\mathbbm {b}_{\ell }) {{\mathscr {G}}_{m}}(\mathbbm {b}_{i-1},\mathbbm {b}_{i}){{\mathscr {G}}_{m}} (\mathbbm {b}_{i},\mathbbm {b}_{\ell })\bigg ]\\&\ \ \ \ \ \ +\Bigg [1+\vartheta ({\mathfrak {a}}_{n},{\mathfrak {a}}_{m})\gamma ^{n}\bigg [ \sum _{i=1}^{\ell }{{\mathscr {G}}_{m}}(\mathbbm {b}_{i-1},\mathbbm {b}_{i}) +\sum _{i=1}^{\ell -1}\vartheta ({\mathscr {U}}^{n}\mathbbm {b}_{i-1},{\mathscr {U}}^{n}\mathbbm {b}_{\ell }) {{\mathscr {G}}_{m}}(\mathbbm {b}_{i-1},\mathbbm {b}_{i}){{\mathscr {G}}_{m}} (\mathbbm {b}_{i},\mathbbm {b}_{\ell })\bigg ]\Bigg ]\\&\ \ \ \ \ \ \times {{\mathscr {G}}_{m}}({\mathfrak {a}}_{n+1},{\mathfrak {a}}_{m}), \ \text {for all} \ n,m\in {\mathbb {N}}, \text {and} \ m>n, \end{aligned}$$

where,

$$\begin{aligned} {{\mathscr {G}}_{m}}({\mathfrak {a}}_{n+1},{\mathfrak {a}}_{m})&\le {{\mathscr {G}}_{m}}({\mathfrak {a}}_{n+1},{\mathfrak {a}}_{n+2})+{{\mathscr {G}}_{m}}({\mathfrak {a}}_{n+2},{\mathfrak {a}}_{m})\\&\ \ \ \ \ \ +\vartheta ({\mathfrak {a}}_{n+1},{\mathfrak {a}}_{m}) {{\mathscr {G}}_{m}} ({\mathfrak {a}}_{n+1},{\mathfrak {a}}_{n+2})+{{\mathscr {G}}_{m}}({\mathfrak {a}}_{n+2},{\mathfrak {a}}_{m})\\&\le \gamma ^{n+1}\bigg [ \sum _{i=1}^{\ell }{{\mathscr {G}}_{m}}(\mathbbm {b}_{i-1}, \mathbbm {b}_{i})+\sum _{i=1}^{\ell -1}\vartheta ({\mathscr {U}}^{n}\mathbbm {b}_{i-1},{\mathscr {U}}^{n} \mathbbm {b}_{\ell }){{\mathscr {G}}_{m}}(\mathbbm {b}_{i-1},\mathbbm {b}_{i}) {{\mathscr {G}}_{m}}(\mathbbm {b}_{i},\mathbbm {b}_{\ell })\bigg ]\\&\ \ \ \ \ \ +\Bigg [1+\vartheta ({\mathfrak {a}}_{n+1},{\mathfrak {a}}_{m})\gamma ^{n+1} \bigg [ \sum _{i=1}^{\ell }{{\mathscr {G}}_{m}}(\mathbbm {b}_{i-1},\mathbbm {b}_{i} )+\sum _{i=1}^{\ell -1}\vartheta ({\mathscr {U}}^{n}\mathbbm {b}_{i-1},{\mathscr {U}}^{n}\mathbbm {b}_{\ell }) {{\mathscr {G}}_{m}}(\mathbbm {b}_{i-1},\mathbbm {b}_{i}){{\mathscr {G}}_{m}} (\mathbbm {b}_{i},\mathbbm {b}_{\ell })\bigg ]\Bigg ]\\&\ \ \ \ \ \ \times {{\mathscr {G}}_{m}}({\mathfrak {a}}_{n+2},{\mathfrak {a}}_{m}). \end{aligned}$$

Combining the above two inequalities, we get,

$$\begin{aligned} {{\mathscr {G}}_{m}}({\mathfrak {a}}_{n},{\mathfrak {a}}_{m})&\le \gamma ^{n} \bigg [ \sum _{i=1}^{\ell }{{\mathscr {G}}_{m}}(\mathbbm {b}_{i-1},\mathbbm {b}_{i}) +\sum _{i=1}^{\ell -1}\vartheta ({\mathscr {U}}^{n}\mathbbm {b}_{i-1},{\mathscr {U}}^{n}\mathbbm {b}_{\ell }) {{\mathscr {G}}_{m}}(\mathbbm {b}_{i-1},\mathbbm {b}_{i}){{\mathscr {G}}_{m}} (\mathbbm {b}_{i},\mathbbm {b}_{\ell })\bigg ]\\&\ \ \ \ \ \ +\gamma ^{n+1}\bigg [\sum _{i=1}^{\ell }{{\mathscr {G}}_{m}}(\mathbbm {b}_{i-1}, \mathbbm {b}_{i})+\sum _{i=1}^{\ell -1}\vartheta ({\mathscr {U}}^{n}\mathbbm {b}_{i-1},{\mathscr {U}}^{n} \mathbbm {b}_{\ell }){{\mathscr {G}}_{m}}(\mathbbm {b}_{i-1},\mathbbm {b}_{i}) {{\mathscr {G}}_{m}}(\mathbbm {b}_{i},\mathbbm {b}_{\ell })\bigg ]\\&\ \ \ \ \ \ \times \Bigg [1+\vartheta ({\mathfrak {a}}_{n},{\mathfrak {a}}_{m})\gamma ^{n}\bigg [ \sum _{i=1}^{\ell } {{\mathscr {G}}_{m}}(\mathbbm {b}_{i-1},\mathbbm {b}_{i})+\sum _{i=1}^{\ell -1} \vartheta ({\mathscr {U}}^{n}\mathbbm {b}_{i-1},{\mathscr {U}}^{n}\mathbbm {b}_{\ell }) {{\mathscr {G}}_{m}}(\mathbbm {b}_{i-1},\mathbbm {b}_{i}) {{\mathscr {G}}_{m}}(\mathbbm {b}_{i},\mathbbm {b}_{\ell })\bigg ]\Bigg ]\\&\ \ \ \ \ \ +\Bigg [1+\vartheta ({\mathfrak {a}}_{n},{\mathfrak {a}}_{m})\gamma ^{n}\bigg [ \sum _{i=1}^{\ell } {{\mathscr {G}}_{m}}(\mathbbm {b}_{i-1},\mathbbm {b}_{i})+\sum _{i=1}^{\ell -1} \vartheta ({\mathscr {U}}^{n}\mathbbm {b}_{i-1},{\mathscr {U}}^{n}\mathbbm {b}_{\ell }) {{\mathscr {G}}_{m}}(\mathbbm {b}_{i-1},\mathbbm {b}_{i}) {{\mathscr {G}}_{m}}(\mathbbm {b}_{i},\mathbbm {b}_{\ell })\bigg ]\Bigg ]\\&\ \ \ \ \ \ \times \Bigg [1+\vartheta ({\mathfrak {a}}_{n+1},{\mathfrak {a}}_{m})\gamma ^{n+1} \bigg [ \sum _{i=1}^{\ell }{{\mathscr {G}}_{m}}(\mathbbm {b}_{i-1},\mathbbm {b}_{i}) +\sum _{i=1}^{\ell -1}\vartheta ({\mathscr {U}}^{n}\mathbbm {b}_{i-1},{\mathscr {U}}^{n}\mathbbm {b}_{\ell }) {{\mathscr {G}}_{m}}(\mathbbm {b}_{i-1},\mathbbm {b}_{i}){{\mathscr {G}}_{m}} (\mathbbm {b}_{i},\mathbbm {b}_{\ell })\bigg ]\Bigg ]\\&\ \ \ \ \ \ \times {{\mathscr {G}}_{m}}({\mathfrak {a}}_{n+2},{\mathfrak {a}}_{m}) \end{aligned}$$

Continuing the same process, we get,

$$\begin{aligned} {{\mathscr {G}}_{m}}({\mathfrak {a}}_{n},{\mathfrak {a}}_{m})&\le \gamma ^{n}\bigg [ \sum _{i=1}^{\ell } {{\mathscr {G}}_{m}}(\mathbbm {b}_{i-1},\mathbbm {b}_{i})+\sum _{i=1}^{\ell -1}\vartheta ({\mathscr {U}}^{n}\mathbbm {b}_{i-1},{\mathscr {U}}^{n}\mathbbm {b}_{\ell }){{\mathscr {G}}_{m}} (\mathbbm {b}_{i-1},\mathbbm {b}_{i}){{\mathscr {G}}_{m}}(\mathbbm {b}_{i},\mathbbm {b}_{\ell })\bigg ]\\&\ \ \ \ \ \ +\gamma ^{n+1}\bigg [\sum _{i=1}^{\ell }{{\mathscr {G}}_{m}}(\mathbbm {b}_{i-1}, \mathbbm {b}_{i})+\sum _{i=1}^{\ell -1}\vartheta ({\mathscr {U}}^{n}\mathbbm {b}_{i-1},{\mathscr {U}}^{n} \mathbbm {b}_{\ell }){{\mathscr {G}}_{m}}(\mathbbm {b}_{i-1},\mathbbm {b}_{i}) {{\mathscr {G}}_{m}}(\mathbbm {b}_{i},\mathbbm {b}_{\ell })\bigg ]\\&\ \ \ \ \ \ \times \Bigg [1+\vartheta ({\mathfrak {a}}_{n},{\mathfrak {a}}_{m})\gamma ^{n}\bigg [ \sum _{i=1}^{\ell } {{\mathscr {G}}_{m}}(\mathbbm {b}_{i-1},\mathbbm {b}_{i})+\sum _{i=1}^{\ell -1} \vartheta ({\mathscr {U}}^{n}\mathbbm {b}_{i-1},{\mathscr {U}}^{n}\mathbbm {b}_{\ell }) {{\mathscr {G}}_{m}}(\mathbbm {b}_{i-1},\mathbbm {b}_{i}) {{\mathscr {G}}_{m}}(\mathbbm {b}_{i},\mathbbm {b}_{\ell })\bigg ]\Bigg ]\\&\ \ \ \ \ \ +\gamma ^{n+2}\bigg [ \sum _{i=1}^{\ell }{{\mathscr {G}}_{m}}(\mathbbm {b}_{i-1}, \mathbbm {b}_{i})+\sum _{i=1}^{\ell -1}\vartheta ({\mathscr {U}}^{n}\mathbbm {b}_{i-1},{\mathscr {U}}^{n} \mathbbm {b}_{\ell }){{\mathscr {G}}_{m}}(\mathbbm {b}_{i-1},\mathbbm {b}_{i}) {{\mathscr {G}}_{m}}(\mathbbm {b}_{i},\mathbbm {b}_{\ell })\bigg ]\\&\ \ \ \ \ \ \times \Bigg [1+\vartheta ({\mathfrak {a}}_{n},{\mathfrak {a}}_{m})\gamma ^{n} \bigg [ \sum _{i=1}^{\ell }{{\mathscr {G}}_{m}}(\mathbbm {b}_{i-1},\mathbbm {b}_{i}) +\sum _{i=1}^{\ell -1}\vartheta ({\mathscr {U}}^{n}\mathbbm {b}_{i-1},{\mathscr {U}}^{n}\mathbbm {b}_{\ell }) {{\mathscr {G}}_{m}}(\mathbbm {b}_{i-1},\mathbbm {b}_{i}){{\mathscr {G}}_{m}} (\mathbbm {b}_{i},\mathbbm {b}_{\ell })\bigg ]\Bigg ]\\&\ \ \ \ \ \ \times \Bigg [1+\vartheta ({\mathfrak {a}}_{n+1},{\mathfrak {a}}_{m})\gamma ^{n+1} \bigg [ \sum _{i=1}^{\ell }{{\mathscr {G}}_{m}}(\mathbbm {b}_{i-1},\mathbbm {b}_{i}) +\sum _{i=1}^{\ell -1}\vartheta ({\mathscr {U}}^{n}\mathbbm {b}_{i-1},{\mathscr {U}}^{n}\mathbbm {b}_{\ell }) {{\mathscr {G}}_{m}}(\mathbbm {b}_{i-1},\mathbbm {b}_{i}){{\mathscr {G}}_{m}} (\mathbbm {b}_{i},\mathbbm {b}_{\ell })\bigg ]\Bigg ]\\&\ \ \ \ \ \ +\cdots +\gamma ^{m-1}\bigg [ \sum _{i=1}^{\ell }{{\mathscr {G}}_{m}} (\mathbbm {b}_{i-1},\mathbbm {b}_{i})+\sum _{i=1}^{\ell -1}\vartheta ({\mathscr {U}}^{n} \mathbbm {b}_{i-1},{\mathscr {U}}^{n}\mathbbm {b}_{\ell }){{\mathscr {G}}_{m}} (\mathbbm {b}_{i-1},\mathbbm {b}_{i}){{\mathscr {G}}_{m}}(\mathbbm {b}_{i},\mathbbm {b}_{\ell })\bigg ]\\&\ \ \ \ \ \ \times \Bigg [1+\vartheta ({\mathfrak {a}}_{n},{\mathfrak {a}}_{m})\gamma ^{n}\bigg [ \sum _{i=1}^{\ell } {{\mathscr {G}}_{m}}(\mathbbm {b}_{i-1},\mathbbm {b}_{i})+\sum _{i=1}^{\ell -1} \vartheta ({\mathscr {U}}^{n}\mathbbm {b}_{i-1},{\mathscr {U}}^{n}\mathbbm {b}_{\ell }) {{\mathscr {G}}_{m}}(\mathbbm {b}_{i-1},\mathbbm {b}_{i}) {{\mathscr {G}}_{m}}(\mathbbm {b}_{i},\mathbbm {b}_{\ell })\bigg ]\Bigg ]\\&\ \ \ \ \ \ \times \Bigg [1+\vartheta ({\mathfrak {a}}_{n+1},{\mathfrak {a}}_{m})\gamma ^{n+1} \bigg [ \sum _{i=1}^{\ell }{{\mathscr {G}}_{m}}(\mathbbm {b}_{i-1}, \mathbbm {b}_{i})+\sum _{i=1}^{\ell -1}\vartheta ({\mathscr {U}}^{n}\mathbbm {b}_{i-1}, {\mathscr {U}}^{n}\mathbbm {b}_{\ell }){{\mathscr {G}}_{m}}(\mathbbm {b}_{i-1}, \mathbbm {b}_{i}){{\mathscr {G}}_{m}}(\mathbbm {b}_{i},\mathbbm {b}_{\ell })\bigg ]\Bigg ]\\&\ \ \ \ \ \ \times \cdots \times \Bigg [1+\vartheta ({\mathfrak {a}}_{m-2},{\mathfrak {a}}_{m}) \gamma ^{m-2}\bigg [ \sum _{i=1}^{\ell }{{\mathscr {G}}_{m}}(\mathbbm {b}_{i-1}, \mathbbm {b}_{i})+\sum _{i=1}^{\ell -1}\vartheta ({\mathscr {U}}^{n}\mathbbm {b}_{i-1}, {\mathscr {U}}^{n}\mathbbm {b}_{\ell }){{\mathscr {G}}_{m}}(\mathbbm {b}_{i-1}, \mathbbm {b}_{i}){{\mathscr {G}}_{m}}(\mathbbm {b}_{i},\mathbbm {b}_{\ell })\bigg ]\Bigg ]\\&\le \gamma ^{n}\bigg [ \sum _{i=1}^{\ell }{{\mathscr {G}}_{m}}(\mathbbm {b}_{i-1}, \mathbbm {b}_{i})+\sum _{i=1}^{\ell -1}\vartheta ({\mathscr {U}}^{n}\mathbbm {b}_{i-1},{\mathscr {U}}^{n} \mathbbm {b}_{\ell }){{\mathscr {G}}_{m}}(\mathbbm {b}_{i-1},\mathbbm {b}_{i}) {{\mathscr {G}}_{m}}(\mathbbm {b}_{i},\mathbbm {b}_{\ell })\bigg ]\\&\ \ \ \ \ \ \times \sum _{i=0}^{m-n-1}\gamma ^{i}\prod _{j=0}^{i-1}\Bigg [1+\vartheta ({\mathfrak {a}}_{n+j}, {\mathfrak {a}}_{m})\gamma ^{n+j}\bigg [ \sum _{i=1}^{\ell }{{\mathscr {G}}_{m}}(\mathbbm {b}_{i-1},\mathbbm {b}_{i})\\&\ \ \ \ \ \ \ \ \ \ \ \ \ \ \ \ \ \ \ \ \ \ \ \ \ \ \ \ \ \ \ \ \ \ \ \ \ \ \ \ \ \ \ +\sum _{i=1}^{\ell -1}\vartheta ({\mathscr {U}}^{n}\mathbbm {b}_{i-1},{\mathscr {U}}^{n}\mathbbm {b}_{\ell }) {{\mathscr {G}}_{m}}(\mathbbm {b}_{i-1},\mathbbm {b}_{i}){{\mathscr {G}}_{m}} (\mathbbm {b}_{i},\mathbbm {b}_{\ell })\bigg ]\Bigg ].\\ \end{aligned}$$

Since $\gamma \in [0,1)$, one can easily prove that the series $\sum _{i=0}^{\infty }{\mathcal {S}}_{i}$ converges as per ratio test, where

$$\begin{aligned} {\mathcal {S}}_{i}=\gamma ^{i}\prod _{j=0}^{i-1}\Bigg [1+\vartheta ({\mathfrak {a}}_{n+j}, {\mathfrak {a}}_{m})\gamma ^{n+j}\bigg [ \sum _{i=1}^{\ell }{{\mathscr {G}}_{m}}(\mathbbm {b}_{i-1}, \mathbbm {b}_{i}) +\sum _{i=1}^{\ell -1}\vartheta ({\mathscr {U}}^{n}\mathbbm {b}_{i-1}, {\mathscr {U}}^{n}\mathbbm {b}_{\ell }){{\mathscr {G}}_{m}}(\mathbbm {b}_{i-1}, \mathbbm {b}_{i}){{\mathscr {G}}_{m}}(\mathbbm {b}_{i},\mathbbm {b}_{\ell })\bigg ]\Bigg ].\\ \end{aligned}$$

Hence, we deduce that ${{\mathscr {G}}_{m}}({\mathfrak {a}}_{n},{\mathfrak {a}}_{m})\rightarrow 0$ as $n,m\rightarrow \infty$, which yields that the sequence $\{{\mathfrak {a}}_{n}\}$ is Cauchy. Since ${\mathscr {M}}$ is complete, subsequently suggests that the sequence $\{{\mathfrak {a}}_{n}\}$ converges in ${\mathscr {M}}$ and from Definition 12, i.e., ${\mathscr {G}}^{\star }$ gratifies the property-$({\textbf{P}}^{\star })$, there is ${\mathfrak {a}}^{\star }\in {\mathscr {M}}$ and $n_{0}\in {\mathbb {N}}$ such that $({\mathfrak {a}}_{n},{\mathfrak {a}}^{\star })\in {\mathcal {E}}({\mathscr {G}}^{\star })$ or $({\mathfrak {a}}^{\star },{\mathfrak {a}}_{n})\in {\mathcal {E}}({\mathscr {G}}^{\star })$ for all $n>n_{0}$ and $\lim _{n\rightarrow \infty }{{\mathscr {G}}_{m}}({\mathfrak {a}}_{n},{\mathfrak {a}}^{\star })=0,$ which shows that $\{{\mathfrak {a}}_{n}\}$ converges to ${\mathfrak {a}}^{\star }$.

By using the assertion ${\mathcal {C}}_{2}$ and inequality 2 when $({\mathfrak {a}}_{n},{\mathfrak {a}}^{\star })\in {\mathcal {E}}({\mathscr {G}}^{\star })$ then we have,

$$\begin{aligned} {{\mathscr {G}}_{m}}({\mathfrak {a}}_{n+1},{\mathscr {U}}{\mathfrak {a}}^{\star })&= {{\mathscr {G}}_{m}}({\mathscr {U}}{\mathfrak {a}}_{n},{\mathscr {U}}{\mathfrak {a}}^{\star })\\&\le \gamma {{\mathscr {G}}_{m}}({\mathfrak {a}}_{n},{\mathfrak {a}}^{\star }) \ \ \text {for all} \ n>n_{0}. \end{aligned}$$

That is, $\lim _{n\rightarrow \infty }{{\mathscr {G}}_{m}}({\mathfrak {a}}_{n+1},{\mathscr {U}}{\mathfrak {a}}^{\star })=0,$ this gives $\{{\mathfrak {a}}_{n}\}$ converges to both ${\mathfrak {a}}^{\star }$ and ${\mathscr {U}}{\mathfrak {a}}^{\star }$. Thus the ${\mathscr {U}}$-Picard sequence $\{{\mathfrak {a}}_{n}\}$ having initial value ${\mathfrak {a}}_{0}$ and it is converges to ${\mathfrak {a}}^{\star }$ and ${\mathscr {U}}{\mathfrak {a}}^{\star }$. As ${\mathfrak {a}}^{\star }\in {\mathscr {M}}$ and ${\mathscr {U}}{\mathfrak {a}}^{\star }\in {\mathscr {U}}({\mathscr {M}})$, this gives ${\mathscr {U}}{\mathfrak {a}}^{\star }={\mathfrak {a}}^{\star }$ as per property $({\textbf {P}})$. This shows that ${\mathfrak {a}}^{\star }$ is a fixed point ${\mathscr {U}}$.

A collection that includes all fixed-points associated with the mapping ${\mathscr {U}}$ can be represented as $Fix({\mathscr {U}})$, therefore we employ the form of the notation ${\mathscr {M}}_{{\mathscr {U}}}$ specified as:

$$\begin{aligned} {\mathscr {M}}_{{\mathscr {U}}}=\{{\mathfrak {a}}\in {\mathscr {M}}:({\mathfrak {a}},{\mathscr {U}}{\mathfrak {a}})\in {\mathcal {E}}({\mathscr {G}}^{\star })\}. \end{aligned}$$

### Example 14

Take ${\mathscr {M}}=\{0\}\sqcup \{\frac{1}{2},\frac{1}{4},\frac{1}{8},\cdots \}$. ${\mathscr {G}}^{\star }={\mathscr {G}}$ is a graph specified with ${\mathscr {V}}({\mathscr {G}})={\mathscr {M}}$ and ${\mathcal {E}}({\mathscr {G}})=\{({\mathfrak {a}},\mathbbm {b})\in {\mathscr {M}}\times {\mathscr {M}}/{\mathfrak {a}}\le \mathbbm {b}\}$. Define a mapping ${{\mathscr {G}}_{m}}:{\mathscr {M}}\times {\mathscr {M}}\rightarrow [0,\infty )$ by

$$\begin{aligned} {{\mathscr {G}}_{m}}({\mathfrak {a}},\mathbbm {b})= {\left\{ \begin{array}{ll} 0, \ \ \ \ \ \ \ \ \ \ \ \text {if} \ \ \ \ {\mathfrak {a}}=\mathbbm {b} \\ |{\mathfrak {a}}-\mathbbm {b}|, \ \text {if} \ \ \ \ {\mathfrak {a}}\ne \mathbbm {b}. \\ \end{array}\right. } \end{aligned}$$

Clearly ${{\mathscr {G}}_{m}}$ is an extended graphical suprametric space on ${\mathscr {M}}$ where $\vartheta :{\mathscr {M}}\times {\mathscr {M}}\rightarrow [1,\infty )$ specified with $\vartheta ({\mathfrak {a}},\mathbbm {b})=e ^{{\mathfrak {a}}+\mathbbm {b}}$ for all ${\mathfrak {a}},\mathbbm {b}\in {\mathscr {M}}$. Let ${\mathscr {U}}:{\mathscr {M}}\rightarrow {\mathscr {M}}$ be a mapping given by ${\mathscr {U}}({\mathfrak {a}})=\frac{{\mathfrak {a}}}{2}$, for all ${\mathfrak {a}}\in {\mathscr {M}}$. Let ${\mathfrak {a}}_{0}=\frac{1}{2}$ such that ${\mathscr {U}}\left( \frac{1}{2}\right) =\left[ \frac{1}{2}\right] _{{\mathscr {G}}^{\star }}^{\ell }$ and the supra-graphical contraction for $\gamma =\frac{1}{2}$, consequently ${\mathscr {U}}$ is a supra-graphical contraction for $\gamma =\frac{1}{2}$ on ${\mathscr {U}}$. It is quite clear that “0” is the unique fixed-point and all of the requirements of Theorem 13 are satisfied.

**Figure 6:**
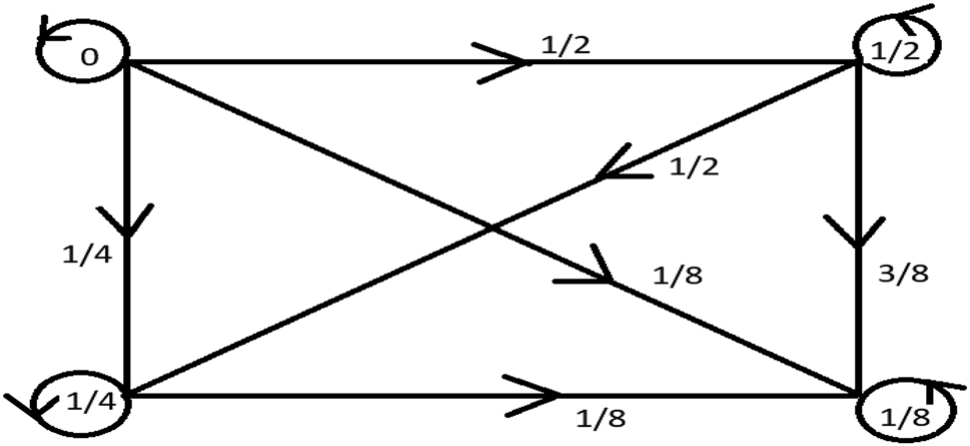
A weighted graph for n = 4.

Above Fig. 6 is a weighted graph for $n=4$ which demonstrates the above Example 14

**Figure 7:**
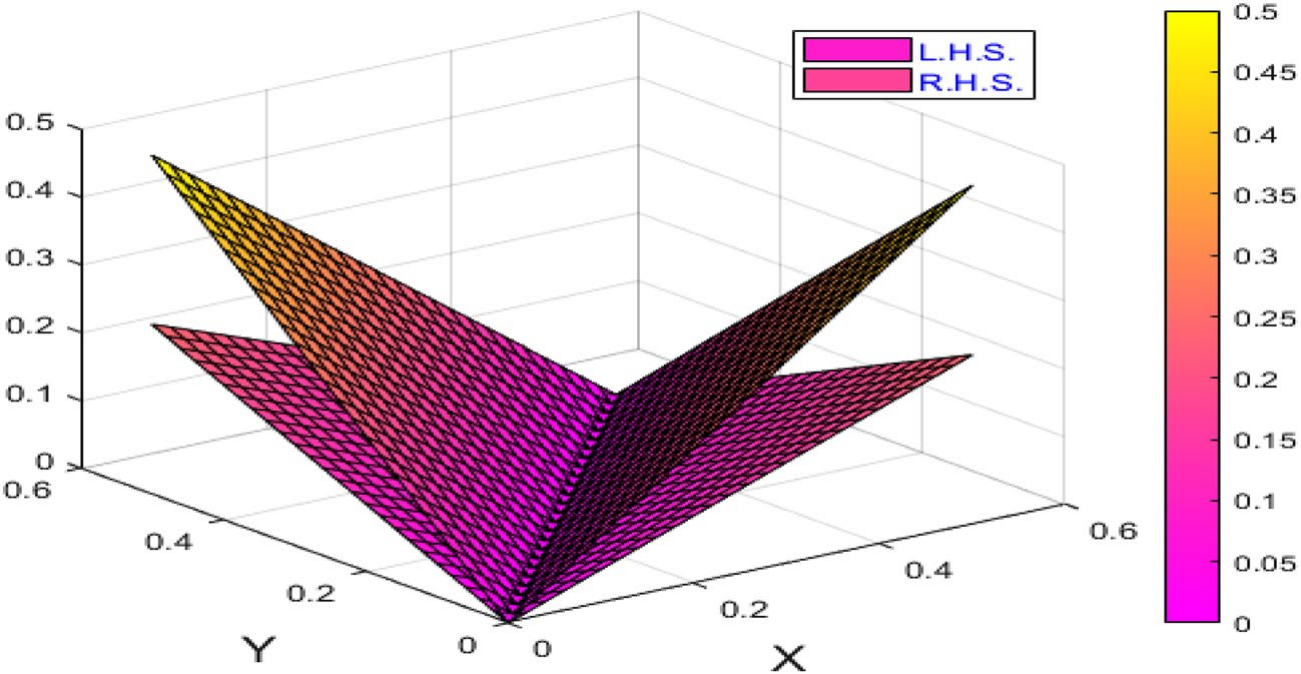
Plot for the corresponding value of the variance between the left and right sides of Example 14 of Theorem 13.

The corresponding value of the variance between the left and right sides of Example 14 of Theorem 13 is as shown in Fig. 7.

Now we prove that supra-graphical contraction is continuous through an example.

### Example 15

Let $({\mathscr {M}},{{\mathscr {G}}_{m}})$ be an extended graphical suprametric space as defined in Example 14. Let ${\mathscr {U}}:{\mathscr {M}}\rightarrow {\mathscr {M}}$ is a supra-graphical contraction. Then ${\mathscr {U}}$ is continuous.

Let $\{{\mathfrak {a}}_{n}\}=\frac{1}{2^{n}}$ be a sequence in ${\mathscr {M}}$ for all $n\in {\mathbb {N}}$. We have $\lim _{n\rightarrow \infty }{{\mathscr {G}}_{m}}\big (\frac{1}{2^{n}},0\big )=\lim _{n\rightarrow \infty } {{\mathscr {G}}_{m}}\big |\frac{1}{2^{n}}-0\big |\rightarrow 0$.

Now consider, $\lim _{n\rightarrow \infty }{{\mathscr {G}}_{m}}\big ({\mathscr {U}}\big (\frac{1}{2^{n}}\big ), {\mathscr {U}}(0)\big )=\lim _{n\rightarrow \infty }{{\mathscr {G}}_{m}}\big |\frac{1}{2^{n+1}}-0\big |\rightarrow 0$. Thus, as “0” is a limit of the sequence $\{\frac{1}{2^{n}}\}$ , we have ${\mathscr {U}}(0)$ is a limit of the sequence $\{{\mathscr {U}}\big (\frac{1}{2^{n}}\big )\}$.

### Corollary 16

Assume that $({\mathscr {M}},{{\mathscr {G}}_{m}})$ be a complete graphical suprametric space and ${\mathscr {U}}:{\mathscr {M}}\rightarrow {\mathscr {M}}$ be a supra-graphical contraction on ${\mathscr {M}}$. Consider that the subsequent assertions are true:

$${\mathscr {G}}_{1}.$$
There exists ${\mathfrak {a}}_{0}\in {\mathscr {M}}$ such that ${\mathscr {U}}{\mathfrak {a}}_{0}\in [{\mathfrak {a}}_{0}]_{{\mathscr {G}}^{\star }}^{\ell }$, where $\ell$ should be in ${\mathbb {N}}.$
$${\mathscr {G}}_{2}.$$
The graph ${\mathscr {G}}^{\star }$ gratifies the property $({\textbf{P}}^{\star })$, and ${\mathfrak {a}}^{\star }\in {\mathscr {M}}$ in such a way that the ${\mathscr {U}}$-Picard sequence $\{{\mathfrak {a}}_{n}\}$ with ${\mathfrak {a}}_{0}\in {\mathscr {M}}$ is ${\mathscr {G}}^{\star }$-termwise connected. Furthermore, it is converges to ${\mathfrak {a}}^{\star }$ and ${\mathscr {U}}{\mathfrak {a}}^{\star }$.
$${\mathscr {G}}_{3}.$$
The quadruple $({\mathscr {M}},{{\mathscr {G}}_{m}},{\mathscr {G}}^{\star },{\mathscr {U}})$ satisfies the property $({\textbf{P}})$, i.e., if ${\mathscr {G}}^{\star }$-termwise connected ${\mathscr {U}}$-Picard sequence $\{{\mathfrak {a}}_{n}\}$ having limits ${\mathfrak {a}}^{\star },\mathbbm {b}^{\star }$, in which ${\mathfrak {a}}^{\star }\in {\mathscr {M}}$ and $\mathbbm {b}^{\star }\in {\mathscr {U}}({\mathscr {M}})$, which implies ${\mathfrak {a}}^{\star }=\mathbbm {b}^{\star }$.

As a result, ${\mathscr {U}}$ has a fixed point.

## Applying graphical structures in automic control systems

**Figure 8:**
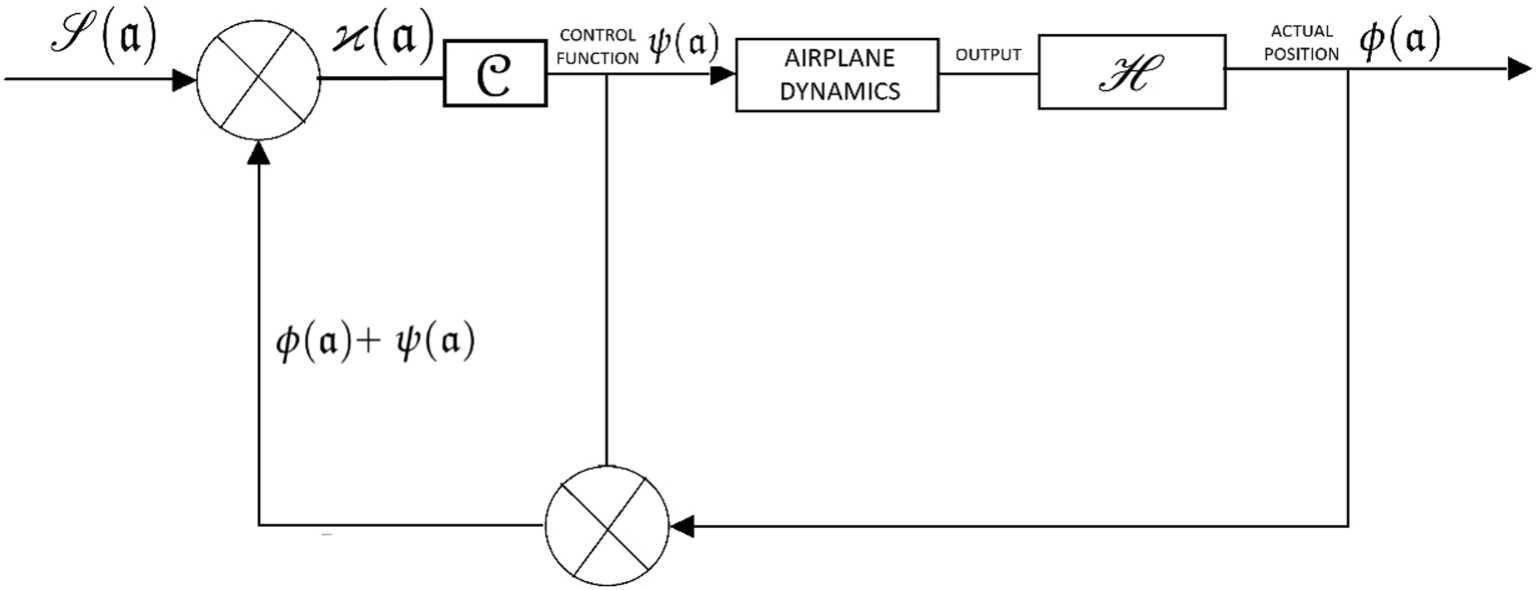
Plot for an automic flight-path generator block.

Take an airplane that carefully follows a piece of terrain as a prime instance. To keep things simple, we should simply consider a couple of variables, namely the horizontal distance and height of flight, to ensure that the airplane flies according to its pitch zone. Both a radar altimeter and a radar will be there as well monitoring the landscape up ahead. There will be certain limitations on aircraft acceleration, which are merely restrictions on the radius of the flight path’s curvature at specific speeds. The most permissible slope of the flight to the horizontal might also have an upper bound. It is hypothetically feasible to create the necessary flight route for the airplane using this information in conjunction with a specified target clearance for the ground. It will be presumed that the work was successfully accomplished and that the necessary flight path ${\mathscr {S}}({\mathfrak {a}})$ is known to be a function of horizontal distance, ${\mathfrak {a}}$. Then, take into account the issue of automatically producing the flight path. Figure 8 demonstrates the system’s block diagram. If the box labeled “${\mathcal {C}}$” is taken to be a unit of storage and delay, it is important to take note of it.

The configuration of the flight controls, which includes the signal for the elevator control, is commonly known as the control function ${\mathscr {S}}({\mathfrak {a}})$. The function $\varphi ({\mathfrak {a}})$ depicts the flight path as the consequence of the control $\psi ({\mathfrak {a}})$. Therefore, the position of the angle in relation to the horizontal throughout the flight path can potentially be represented by $\varphi ({\mathfrak {a}})$. As indicated in the figure, the precise airplane location $\phi ({\mathfrak {a}})$ has been included to $\psi ({\mathfrak {a}})$ and given right back for verification to determine the necessary route ${\mathscr {S}}({\mathfrak {a}})$. The signal $\varkappa ({\mathfrak {a}})$ is then generated and held in the box denoted by ${\mathcal {C}}$ unless it gets released and turns into the newly created control function $\psi ({\mathfrak {a}})$.

To distinguish between sequentially created functions $\psi ({\mathfrak {a}})$, the latter is going to be assigned a suffix *i*.

The system will function in such a way that starting with an arbitrary control function $\psi _{0}({\mathfrak {a}})$, the necessary control function $\psi ({\mathfrak {a}})$ will eventually be developed immediately at this point:

$$\begin{aligned} \psi _{i+1}({\mathfrak {a}})=\varkappa _{i}({\mathfrak {a}})=-{\mathscr {S}}({\mathfrak {a}})+\psi _{i}({\mathfrak {a}})+\phi _{i}({\mathfrak {a}}). \end{aligned}$$

Since

3 $$\begin{aligned} \phi _{i}({\mathfrak {a}})={\mathscr {H}}_{\varphi _{i}}({\mathfrak {a}})={\mathscr {H}}{\mathcal {A}}\psi _{i}({\mathfrak {a}}), \end{aligned}$$

then

4 $$\begin{aligned} \psi _{i-1}({\mathfrak {a}})=\varkappa _{i}({\mathfrak {a}})=-{\mathscr {S}}({\mathfrak {a}})+({\mathscr {H}}{\mathcal {A}} +{\mathcal {I}})\psi _{i}({\mathfrak {a}}). \end{aligned}$$

The right-hand side of the expression must be a supra-graphical contractive mapping in order to allow the recursive procedure outlined in Eq. (4) to reach convergence. The solution is then

$$\begin{aligned}{} & {} \psi ({\mathfrak {a}})=-{\mathscr {S}}({\mathfrak {a}})+({\mathscr {H}}{\mathcal {A}}+{\mathcal {I}})\psi ({\mathfrak {a}}),\\ \end{aligned}$$

That is, ${\mathscr {S}}({\mathfrak {a}})={\mathscr {H}}{\mathcal {A}}\psi ({\mathfrak {a}})=\phi ({\mathfrak {a}})$, from Eq. (3).

In the context of simple terms, the control function is set up to achieve the intended outcome, namely that the real position $\phi ({\mathfrak {a}})$ meets the appropriate trajectory ${\mathscr {S}}({\mathfrak {a}})$.

Consider ${\mathscr {M}}=C([0,{\mathbb {T}}],{\mathbb {R}})$, set of all functions which are real-valued and continuous on $[0,{\mathbb {T}}]$ and ${\mathscr {D}}=\{\psi ({\mathfrak {a}})\in {\mathscr {M}}/{\mathfrak {a}}\in [0,{\mathbb {T}}]\}.$

Establish the graph ${\mathscr {G}}$ and ${\mathscr {G}}^{\star }$ by ${\mathscr {G}}={\mathscr {G}}^{\star }, \ {\mathscr {V}}({\mathscr {G}})={\mathscr {M}}$ and

$$\begin{aligned} {\mathcal {E}}({\mathscr {G}})=\{({\mathscr {K}},{\mathscr {L}})\in {\mathscr {M}}\times {\mathscr {M}}/{\mathscr {K}},{\mathscr {L}}\in {\mathscr {D}}, \ {\mathscr {K}}({\mathfrak {a}})\le {\mathscr {L}}({\mathfrak {a}}), \text {for all} \ {\mathfrak {a}}\in [0,{\mathbb {T}}]\}. \end{aligned}$$

Evidently, the pair $({\mathscr {M}},{{\mathscr {G}}_{m}})$ is an extended graphical suprametric space and ${{\mathscr {G}}_{m}}:{\mathscr {M}}\times {\mathscr {M}}\rightarrow {\mathbb {R}}$ specified with ${{\mathscr {G}}_{m}}^{\omega }({\mathscr {K}},{\mathscr {L}})=\xi ({\mathscr {K}}, {\mathscr {L}})[\xi ({\mathscr {K}},{\mathscr {L}})+\omega ]$; where $\xi ({\mathscr {K}},{\mathscr {L}})$ is a graphical metric and $\omega$ is a positive real number, for all ${\mathscr {K}},{\mathscr {L}}\in {\mathscr {M}}$. Obviously $({\mathscr {M}},{{\mathscr {G}}_{m}})$ is complete extended graphical suprametric space. Note that $\xi ({\mathscr {K}},{\mathscr {L}})=|{\mathscr {K}}-{\mathscr {L}}|$.

Allow the plane to fly with an identical speed ${\mathscr {W}}$. Assuming a flight angle $\phi ({\mathfrak {a}})$, the height $\varphi ({\mathfrak {a}})$ over the datum corresponds to follows:

5 $$\begin{aligned} \phi ({\mathfrak {a}})=\phi ({\mathfrak {a}}_{0})+{\mathscr {W}}\int \limits _{{\mathfrak {a}}_{0}}^{{\mathfrak {a}}}tan\varphi d{\mathfrak {a}}={\mathscr {H}}\varphi ({\mathfrak {a}}). \end{aligned}$$

The following formula can be used to model the airplane dynamics after appropriate streamlined features,

6 $$\begin{aligned} \frac{d^{3}\varphi }{d\lambda ^{3}}+\alpha _{2}\frac{d^{2}\varphi }{d\lambda ^{2}} +\alpha _{1}\frac{d\varphi }{d\lambda }=\beta _{2}\frac{d^{2}\psi }{d\lambda ^{2}}+\beta _{1}\frac{d\psi }{d\lambda }-\beta _{0}\psi . \end{aligned}$$

Each prefix *i* within the recursive scheme is valid in this instance.

Assume that $\rho$ and $\sigma$ are the equation’s roots.

$$\begin{aligned} p^{2}+\alpha _{2}p+\alpha _{1}=0, \end{aligned}$$

and put

$$\begin{aligned} \Psi \{{\mathfrak {a}}(\lambda )\}\equiv \beta _{2}\frac{d^{2}\psi }{d\lambda ^{2}}+\beta _{1}\frac{d\psi }{d\lambda }-\beta _{0}\psi . \end{aligned}$$

For a given speed, ${\mathfrak {a}}$ is a function of $\lambda$ and hence $\varphi ,\psi$ are functions either of ${\mathfrak {a}}$ or of $\lambda$.

Then, from Eq. (6),

7 $$\begin{aligned} \varphi \{{\mathfrak {a}}(\lambda )\}&=\varphi ({\mathfrak {a}}_{0})+\int \limits _{\lambda _{0}}^{\lambda }{} e ^{\sigma \lambda _{1}} \int \limits _{\lambda _{0}}^{\lambda _{1}}{} e ^{-\sigma \lambda _{2}}{} e ^{\rho \lambda _{2}}\int \limits _{\lambda _{0}}^{\lambda _{2}}{} e ^{-\rho \lambda _{3}}\Psi \{{\mathfrak {a}}(\lambda _{3})\}d\lambda _{3}d\lambda _{2}d\lambda _{1}\\&\equiv {\mathcal {A}}\psi {{\mathfrak {a}}}.\nonumber \end{aligned}$$

Thus, ${\mathcal {A}}$ and ${\mathscr {H}}$ in Eq. (4) are specified.

Let the function ${\mathscr {H}}{\mathcal {A}}+{\mathcal {I}}:{\mathscr {M}}\rightarrow {\mathscr {M}}$ by $({\mathscr {H}}{\mathcal {A}}+{\mathcal {I}})(\psi _{i}({\mathfrak {a}}))={\mathscr {H}}{\mathcal {A}}\psi _{i}({\mathfrak {a}}) +{\mathcal {I}}\psi _{i}({\mathfrak {a}})$, where ${\mathcal {I}}$ is the identity function from ${\mathscr {M}}$ to ${\mathscr {M}}$.

Now to prove that the control function is in such a way that the real position $\phi ({\mathfrak {a}})$ identical to the ${\mathscr {S}}({\mathfrak {a}})$, we need to prove that the right side of Eq. (4) is supra-graphical contraction. Consider,

$$\begin{aligned}&{{\mathscr {G}}_{m}}[({\mathscr {H}}{\mathcal {A}}+{\mathcal {I}})\psi _{i}({\mathfrak {a}}), ({\mathscr {H}}{\mathcal {A}}+{\mathcal {I}})\psi _{i-1}({\mathfrak {a}})]\\&\quad =\xi [({\mathscr {H}}{\mathcal {A}}+{\mathcal {I}})\psi _{i}({\mathfrak {a}}),({\mathscr {H}}{\mathcal {A}} +{\mathcal {I}})\psi _{i-1}({\mathfrak {a}})]\big [\xi [({\mathscr {H}}{\mathcal {A}}+{\mathcal {I}})\psi _{i} ({\mathfrak {a}}),({\mathscr {H}}{\mathcal {A}}+{\mathcal {I}})\psi _{i-1}({\mathfrak {a}})]+\omega \big ]\\&\quad =|({\mathscr {H}}{\mathcal {A}}+{\mathcal {I}})\psi _{i}({\mathfrak {a}})-({\mathscr {H}}{\mathcal {A}} +{\mathcal {I}})\psi _{i-1}({\mathfrak {a}})|\big [|({\mathscr {H}}{\mathcal {A}}+{\mathcal {I}})\psi _{i} ({\mathfrak {a}})-({\mathscr {H}}{\mathcal {A}}+{\mathcal {I}})\psi _{i-1}({\mathfrak {a}})|+\omega \big ]\\&\quad =|{\mathscr {H}}{\mathcal {A}}\psi _{i}({\mathfrak {a}})+\psi _{i}({\mathfrak {a}})-{\mathscr {H}} {\mathcal {A}}\psi _{i-1}({\mathfrak {a}})-\psi _{i-1}({\mathfrak {a}})|\big [|{\mathscr {H}}{\mathcal {A}}\psi _{i} ({\mathfrak {a}})+\psi _{i}({\mathfrak {a}})-{\mathscr {H}}{\mathcal {A}}\psi _{i-1}({\mathfrak {a}}) -\psi _{i-1}({\mathfrak {a}})|+\omega \big ]\\&\quad \le \Biggl [ \left[ \frac{{\mathscr {H}}{\mathcal {A}}\psi _{i}({\mathfrak {a}})-{\mathscr {H}}{\mathcal {A}}\psi _{i-1} ({\mathfrak {a}})}{\psi _{i}({\mathfrak {a}})-\psi _{i-1}({\mathfrak {a}})}+1 \right] |\psi _{i}({\mathfrak {a}})-\psi _{i-1}({\mathfrak {a}})|\Biggr ]\\&\qquad \ \ \ \ \ \ \ \ \ \ \ \ \ \times \Biggl [ \left[ \frac{{\mathscr {H}}{\mathcal {A}}\psi _{i}({\mathfrak {a}}) -{\mathscr {H}}{\mathcal {A}}\psi _{i-1}({\mathfrak {a}})}{\psi _{i}({\mathfrak {a}})-\psi _{i-1} ({\mathfrak {a}})}+1 \right] |\psi _{i}({\mathfrak {a}})-\psi _{i-1}({\mathfrak {a}})|+\omega \Biggr ]\\&\quad \le \Bigg | \frac{{\mathscr {H}}{\mathcal {A}}\psi _{i}({\mathfrak {a}})-{\mathscr {H}}{\mathcal {A}}\psi _{i-1} ({\mathfrak {a}})}{\psi _{i}({\mathfrak {a}})-\psi _{i-1}({\mathfrak {a}})} +1 \Bigg |^{2}|\psi _{i}({\mathfrak {a}})-\psi _{i-1}({\mathfrak {a}})|^{2}\\&\qquad \ \ \ \ \ \ \ \ \ \ \ \ \ +\omega \Bigg | \frac{{\mathscr {H}}{\mathcal {A}}\psi _{i}({\mathfrak {a}}) -{\mathscr {H}}{\mathcal {A}}\psi _{i-1}({\mathfrak {a}})}{\psi _{i}({\mathfrak {a}})-\psi _{i-1}({\mathfrak {a}})} +1 \Bigg | |\psi _{i}({\mathfrak {a}})-\psi _{i-1}({\mathfrak {a}})|\\&\quad \le \Bigg | \frac{{\mathscr {H}}{\mathcal {A}}\psi _{i}({\mathfrak {a}}) -{\mathscr {H}}{\mathcal {A}}\psi _{i-1}({\mathfrak {a}})}{\psi _{i}({\mathfrak {a}}) -\psi _{i-1}({\mathfrak {a}})}+1 \Bigg ||\psi _{i}({\mathfrak {a}})-\psi _{i-1}({\mathfrak {a}})|\\&\qquad \ \ \ \ \ \ \ \ \ \ \ \ \ \times \Biggl [\Bigg | \frac{{\mathscr {H}} {\mathcal {A}}\psi _{i}({\mathfrak {a}})-{\mathscr {H}}{\mathcal {A}}\psi _{i-1} ({\mathfrak {a}})}{\psi _{i}({\mathfrak {a}})-\psi _{i-1}({\mathfrak {a}})} \Bigg | |\psi _{i} ({\mathfrak {a}})-\psi _{i-1}({\mathfrak {a}})|+\omega \Biggr ]\\&\qquad < \Bigg | \frac{{\mathscr {H}}{\mathcal {A}}\psi _{i}({\mathfrak {a}}) -{\mathscr {H}}{\mathcal {A}}\psi _{i-1}({\mathfrak {a}})}{\psi _{i} ({\mathfrak {a}})-\psi _{i-1}({\mathfrak {a}})}+1 \Bigg | |\psi _{i} ({\mathfrak {a}})-\psi _{i-1}({\mathfrak {a}})|[|\psi _{i}({\mathfrak {a}})-\psi _{i-1}({\mathfrak {a}})|+\omega ]\\&\quad =\gamma |\psi _{i}({\mathfrak {a}})-\psi _{i-1}({\mathfrak {a}})|[|\psi _{i} ({\mathfrak {a}})-\psi _{i-1}({\mathfrak {a}})|+\omega ]\\&\quad =\gamma \xi (\psi _{i}({\mathfrak {a}}),\psi _{i-1}({\mathfrak {a}}))[\xi (\psi _{i} ({\mathfrak {a}}),\psi _{i-1}({\mathfrak {a}}))+\omega ]\\&\quad =\gamma {{\mathscr {G}}_{m}}(\psi _{i}({\mathfrak {a}}),\psi _{i-1}({\mathfrak {a}})), \end{aligned}$$

where $$\gamma =\Bigg | \frac{{\mathscr {H}}{\mathcal {A}}\psi _{i}({\mathfrak {a}}) -{\mathscr {H}}{\mathcal {A}}\psi _{i-1}({\mathfrak {a}})}{\psi _{i}({\mathfrak {a}})-\psi _{i-1}({\mathfrak {a}})}+1 \Bigg |$$.

Supra-contraction is satisfies if $\gamma =\Bigg | \frac{{\mathscr {H}}{\mathcal {A}}\psi _{i}({\mathfrak {a}}) -{\mathscr {H}}{\mathcal {A}}\psi _{i-1}({\mathfrak {a}})}{\psi _{i}({\mathfrak {a}})-\psi _{i-1}({\mathfrak {a}})}+1 \Bigg |<1$ for all $i,{\mathfrak {a}}$. In other words,

8 $$\begin{aligned} -2<\frac{{\mathscr {H}}{\mathcal {A}}\psi _{i}({\mathfrak {a}})-{\mathscr {H}}{\mathcal {A}}\psi _{i-1} ({\mathfrak {a}})}{\psi _{i}({\mathfrak {a}})-\psi _{i-1}({\mathfrak {a}})}<0, \ \text {for all} \ i,{\mathfrak {a}}. \end{aligned}$$

However, from Eq. (5),

$$\begin{aligned} {\mathscr {H}}{\mathcal {A}}\psi _{i}({\mathfrak {a}})-{\mathscr {H}}{\mathcal {A}}\psi _{i-1}({\mathfrak {a}}) ={\mathscr {W}}\int \limits _{{\mathfrak {a}}_{0}}^{{\mathfrak {a}}}(tan\varphi _{i}-tan\varphi _{i-1})d{\mathfrak {a}}. \end{aligned}$$

9 $$\begin{aligned} \frac{{\mathscr {H}}{\mathcal {A}}\psi _{i}({\mathfrak {a}})-{\mathscr {H}}{\mathcal {A}}\psi _{i-1} ({\mathfrak {a}})}{\psi _{i}({\mathfrak {a}})-\psi _{i-1}({\mathfrak {a}})}&=\frac{{\mathscr {W}}\int \limits _{{\mathfrak {a}}_{0}}^{{\mathfrak {a}}}(tan\varphi _{i} -tan\varphi _{i-1})d{\mathfrak {a}}}{\psi _{i}({\mathfrak {a}})-\psi _{i-1}({\mathfrak {a}})}\nonumber \\&\ge -{\mathscr {W}}m({\mathfrak {a}},{\mathfrak {a}}_{0}). \end{aligned}$$

Since, by taking

10 $$\begin{aligned} \frac{d}{d\psi }(tan\varphi )\ge -m, \end{aligned}$$

where *m* is a positive number.

Then,

$$\begin{aligned} tan\varphi _{i}-tan\varphi _{i-1}&=\int \limits _{\psi _{i-1}}^{\psi _{i}}\frac{d}{d\psi }(tan\varphi )d\psi \\&\ge -m(\psi _{i},\psi _{i-1}). \end{aligned}$$

From inequality 9 and physical considerations, when $\psi ({\mathfrak {a}})$ increases, $\varphi ({\mathfrak {a}})$ decreases and vice versa. We get

$$\begin{aligned} -2<\frac{{\mathscr {H}}{\mathcal {A}}\psi _{i}({\mathfrak {a}})-{\mathscr {H}}{\mathcal {A}}\psi _{i-1} ({\mathfrak {a}})}{\psi _{i}({\mathfrak {a}})-\psi _{i-1}({\mathfrak {a}})}<0. \end{aligned}$$

Thus supra-graphical contraction satisfies for

11 $$\begin{aligned} {\mathfrak {a}}-{\mathfrak {a}}_{0}<\frac{2}{{\mathscr {W}}m}. \end{aligned}$$

It follows that right side of Eq. (4) satisfies supra-graphical contraction.

Hence the solution is,

$$\begin{aligned} {\mathscr {S}}({\mathfrak {a}})={\mathscr {H}}{\mathcal {A}}\psi ({\mathfrak {a}})=\phi ({\mathfrak {a}}). \end{aligned}$$

Therefore, throughout this range, the necessary $\psi ({\mathfrak {a}})$ algorithm may be developed automatically.

To gain a clearer understanding of *m* in inequality 10, it will be noted that

12 $$\begin{aligned} \frac{d(tan\varphi )}{d\psi }&=sec^{2}\varphi \frac{d\varphi }{d\psi }\nonumber \\&=(1+tan^{2}\varphi )\frac{d\varphi }{d\lambda }\frac{d\lambda }{d\psi } \nonumber \\&=\biggl [1+\Bigl (\frac{d\phi }{d{\mathfrak {a}}}\Bigr )^{2}\biggr ]\biggl [\frac{d\varphi /d\lambda }{d\psi /d\lambda }\biggr ]. \end{aligned}$$

The upper limit of $\Bigl (\frac{d\phi }{d{\mathfrak {a}}}\Bigr )^{2}$ is set by the limitations on aircraft acceleration, while $\frac{d\varphi }{d\lambda }$ and $\frac{d\psi }{d\lambda }$ are related through Eq. (6), consisting of the aircraft dynamics. As a result, *m* is associated with identical parameters, and inequality 11 determines ${\mathfrak {a}}-{\mathfrak {a}}_{0}$ for a reasonable value of *m*.

It has already been demonstrated that provided equality 4 is a supra-graphical contractive mapping, a suitable control function is able to be developed automatically. Once this aspect has been accomplished across the range ${\mathfrak {a}}-{\mathfrak {a}}_{0}$ provided by inequality 11, starting from a point ${\mathfrak {a}}_{0}$, a different starting point ${\mathfrak {a}}_{1}$ is able to be identified, and the procedure continued up to the expected $\psi ({\mathfrak {a}})$ is recognized throughout the complete range. The value of $\gamma$ in inequality 2 determines how quickly the recursive procedure in equality 4 converges. The convergence occurs more quickly regardless of the value of $\gamma$. This may be observed in inequality 2 and equality 8 that, $\gamma$ seems correlated with *m* in turn. In fact, $\gamma$ is the value of

$$\begin{aligned} \Bigg | \frac{{\mathscr {H}}{\mathcal {A}}\psi _{i}({\mathfrak {a}})-{\mathscr {H}}{\mathcal {A}}\psi _{i-1} ({\mathfrak {a}})}{\psi _{i}({\mathfrak {a}})-\psi _{i-1}({\mathfrak {a}})}+1 \Bigg |, \end{aligned}$$

in the expression,

$$\begin{aligned} \Bigg | \frac{{\mathscr {H}}{\mathcal {A}}\psi _{i}({\mathfrak {a}})-{\mathscr {H}}{\mathcal {A}}\psi _{i-1} ({\mathfrak {a}})}{\psi _{i}({\mathfrak {a}})-\psi _{i-1}({\mathfrak {a}})}+1 \Bigg ||\psi _{i}({\mathfrak {a}})-\psi _{i-1}({\mathfrak {a}})|. \end{aligned}$$

Hence,

$$\begin{aligned} \gamma \le \Bigg | \frac{{\mathscr {H}}{\mathcal {A}}\psi _{i}({\mathfrak {a}})-{\mathscr {H}}{\mathcal {A}}\psi _{i-1} ({\mathfrak {a}})}{\psi _{i}({\mathfrak {a}})-\psi _{i-1}({\mathfrak {a}})}+1 \Bigg |, \end{aligned}$$

and the closer $\frac{{\mathscr {H}}{\mathcal {A}}\psi _{i}({\mathfrak {a}})-{\mathscr {H}}{\mathcal {A}}\psi _{i-1} ({\mathfrak {a}})}{\psi _{i}({\mathfrak {a}})-\psi _{i-1}({\mathfrak {a}})}$ is to -1, the smaller is $\gamma$. In that case, ${\mathscr {W}}m({\mathfrak {a}}-{\mathfrak {a}}_{0})$ in inequality 11 ideally, be +1 or near to it.

Figure 8 is a automic flight-path generator block diagram. Both of the parameters range ${\mathfrak {a}}-{\mathfrak {a}}_{0}$ and the rate of convergence can be adjusted for one another. It should be emphasized that the need for a graphical supra-contractive mapping determined hereunder depends on the existence of the terrain function, ${\mathscr {S}}({\mathfrak {a}})$. As a result, the necessary control function is able to be generated for many types of terrain using the identical framework and hardware. Due to the “$tan\varphi$” in Eq. (5), the framework that is taken into consideration in relation to the terrain adhering to is non-linear. As previously established, this is not something that restricts the usefulness of graphical supra-contractive mapping. It is not stated that the aforementioned approach is the only one that may be used to resolve the difficulty or that the terrain-following problem has been fully resolved. The graphical supra-contractive mapping, nevertheless, has already been demonstrated to be useful to the automatic process of creating control functions in both nonlinear and linear systems.

Whenever the hardware responds quickly enough across the appropriate range, a method similar to that in Figure 8 could be utilized in an aircraft. It might additionally be employed in structure, modeling, and training studies. The terrain function, ${\mathscr {S}}({\mathfrak {a}})$, unambiguously impacts the control function, $\psi ({\mathfrak {a}})$, so whenever the latter shifts, the former is automatically updated. The control system can be called adaptive in this sense.

## Applying graphical structures in control system optimization

Take into account the following equations for the linear, time-varying system with state vector ${\mathfrak {a}}(\lambda )$ of dimension *n*, output vector $\hbar (\lambda )$ of dimension *r* and, control vector ${\mathfrak {z}}(\lambda )$ of dimension *m*:

13 $$\begin{aligned} \dot{{\mathfrak {a}}}(\lambda )={\mathscr {P}}(\lambda ){\mathfrak {a}}(\lambda )+{\mathscr {Q}}(\lambda ){\mathfrak {z}}(\lambda ) \end{aligned}$$

14 $$\begin{aligned} \hbar (\lambda )={\mathscr {R}}(\lambda ){\mathfrak {a}}(\lambda )+{\mathscr {T}}(\lambda ){\mathfrak {z}}(\lambda ). \end{aligned}$$

${\mathscr {P}}(\lambda )$ is a matrix which constitutes $n\times n$ order, ${\mathscr {Q}}(\lambda )$ is a matrix which constitutes $n\times m$ order, $\hbar (\lambda )$ is a matrix which constitutes $r\times n$ order, and ${\mathscr {T}}(\lambda )$ is a matrix which constitutes $r\times m$ order since all the matrices have compatible properties. Controlling the system to produce an outcome that is reasonably near to the intended result $\hbar _{d}(\lambda )$ is the goal. The method of estimation for error $e (\lambda )$ is as outlined below:

15 $$\begin{aligned} e (\lambda )=\hbar _{d}(\lambda )-\hbar (\lambda ). \end{aligned}$$

Control of energy must be restrained in order to lower the cost functional ${\mathscr {F}}$ while pursuing the goal, which included

16 $$\begin{aligned} {\mathscr {F}}=\int \limits _{\lambda _{0}}^{\texttt {T}}[e ^{\prime }(\lambda )\{\gamma (\lambda ) e (\lambda )\}+{\mathfrak {z}}^{\prime }(\lambda )\{{\mathscr {A}}(\lambda ){\mathfrak {z}}(\lambda )\}]d\lambda . \end{aligned}$$

First consider the potential of impulsive functions in $\gamma (\lambda )$ for the purpose of foreseeing the need for restricting the terminal state and take

17 $$\begin{aligned} \gamma (\lambda )={\mathscr {B}}(\lambda )+{\mathscr {X}}(\lambda ), \end{aligned}$$

where, ${\mathscr {B}}(\lambda )$ be piecewise continuous function. ${\mathscr {X}}(\lambda )$’s components either correspond to impulse functions $\theta _{ij}\psi (\lambda -\texttt {T})$ or zeros. When Eq. (17) is used in place of Eq. (16), the cost function yields,

18 $$\begin{aligned} {\mathscr {F}}=e ^{\prime }(\texttt {T})\{{\mathscr {X}}{} e (\texttt {T})\} +\int \limits _{\lambda _{0}}^{\texttt {T}}[e ^{\prime }(\lambda )\{{\mathscr {B}}(\lambda ) e (\lambda )\}+{\mathfrak {z}}^{\prime }(\lambda )\{{\mathscr {A}}(\lambda ){\mathfrak {z}}(\lambda )\}]d\lambda . \end{aligned}$$

The variables corresponding to $\theta _{ij}$ make up the data elements of the constant matrix ${\mathscr {X}}$ in this instance. Subsequently, it is important to note that the Eq. (16) consequently incorporates the final outcome of control system together with the minimal energy issue-Balakrishna et al.,^23^ looked into this. The primary component of Eq. (18) implicitly takes into consideration the weighting of a terminal state as necessary. The integral Eq. (18), which uses the function ${\mathfrak {z}}(\lambda )$, must now be constrained to a function space that guarantees the control energy’s existence. With regard to the sake of argument, it is taken into account that ${\mathfrak {z}}(\lambda )$ is an element of the *m*-dimensional Hilbert space ${\mathscr {H}}_{m}$ that is characterized by the product space $\{{\mathcal {L}}^{2}(\lambda _{0},\texttt {T})\}_{m}$ of square-integrable functions in the range $[\lambda _{0},\texttt {T}]$ that includes the norm of a parameter ${\mathfrak {z}}(\lambda )$ with the representation of $||{\mathfrak {z}}(\lambda )||$ can be determined by the inner product as follows:

19 $$\begin{aligned} ||{\mathfrak {z}}(\lambda )||=\sqrt{\langle {\mathfrak {z}}(\lambda ),{\mathfrak {z}}(\lambda )\rangle } =\sqrt{\left\{ \int \limits _{\lambda _{0}}^{\texttt {T}}{\mathfrak {z}}^{\prime }(\lambda ){\mathfrak {z}}(\lambda )d\lambda \right\} }. \end{aligned}$$

The notion of “$\langle \varkappa ,{\mathcal {Y}}\rangle =\langle {\mathcal {Y}},\varkappa \rangle$” refers to the inner product of two separate components $\varkappa$ and ${\mathcal {Y}}$ in ${\mathscr {H}}_{m}$ which can be described as follows:

20 $$\begin{aligned} \langle \varkappa ,{\mathcal {Y}}\rangle =\int \limits _{\lambda _{0}}^{\texttt {T}}{\mathcal {Y}}^{\prime }\varkappa d\lambda =\int \limits _{\lambda _{0}}^{\texttt {T}}\varkappa ^{\prime }{\mathcal {Y}} d\lambda . \end{aligned}$$

We need to ensure that $e (\lambda )$ is square-integrable because we have limited our study to functions ${\mathfrak {z}}(\lambda )$ in ${\mathscr {H}}_{m}$ and primarily for the reason we need the whole integral under Eq. (18) to exist. In particular, we insist whether $e (\lambda )$ belongs to the closed range $[\lambda _{0},\texttt {T}]$ of square-integrable functions in the *r*-dimensional Hilbert space ${\mathscr {H}}_{r}$. The procedures suggested under Eqs. (13) and (14) need to transform functions from ${\mathfrak {z}}(\lambda )$ in ${\mathscr {H}}_m$ through functions from $e (\lambda )$ in ${\mathscr {H}}_{r}$ with the aim to satisfy this criterion. To accomplish this, as per emphasized in^24^ and provides the general solution to Eq. (13).

21 $$\begin{aligned} {\mathfrak {a}}(\lambda )=\Psi (\lambda ,\lambda _{0}){\mathfrak {a}}(\lambda _{0}) +\int \limits _{\lambda _{0}}^{\lambda }\Psi (\lambda ,\varsigma ){\mathscr {Q}}(\varsigma ){\mathfrak {z}}(\varsigma )d\varsigma , \end{aligned}$$

where $\Psi (\lambda ,\lambda _{0})$ be a classical transition matrix which gratifies

22 $$\begin{aligned} \Psi (\lambda ,\lambda _{0})={\mathscr {P}}(\lambda )\Psi (\lambda ,\lambda _{0}). \end{aligned}$$

Thus, from Eqs. (14), (15) and (21), we obtain

23 $$\begin{aligned} e (\lambda )=\hbar _{d}(\lambda )-{\mathscr {S}}(\lambda ){\mathfrak {z}}(\lambda ) -{\mathscr {R}}(\lambda )\left\{ \Psi (\lambda ,\lambda _{0}){\mathfrak {a}}(\lambda _{0})+ \int \limits _{\lambda _{0}}^{\lambda }\Psi (\lambda ,\varsigma ){\mathscr {Q}}(\varsigma ){\mathfrak {z}}(\varsigma )d\varsigma \right\} . \end{aligned}$$

Under preconceived notions, the primary component is automatically in ${\mathscr {H}}_{r}$. We determine the second term’s norm to determine its position and the outcomes that follow:

$$\begin{aligned} ||{\mathscr {S}}{\mathfrak {z}}||^{2}=\langle {\mathscr {S}}{\mathfrak {z}},{\mathscr {S}}{\mathfrak {z}}\rangle \le \texttt {max}_{i}{} \texttt {sup}_{\lambda \in [\lambda ,\texttt {T}]}\gamma _{i}(\lambda )\langle {\mathfrak {z}},{\mathfrak {z}}\rangle , \end{aligned}$$

where $\gamma _{i}$ are the eigenvalues of ${\mathscr {S}}^{\prime }(\lambda ),{\mathscr {S}}(\lambda )$. Therefore,

24 $$\begin{aligned} ||{\mathscr {S}}{\mathfrak {z}}||^{2}\le \texttt {max}_{i}{} \texttt {sup}_{\lambda \in [\lambda ,\texttt {T}]}\gamma _{i}(\lambda )||{\mathfrak {z}}||^{2}, \end{aligned}$$

and provided that

$$\begin{aligned} \texttt {max}_{i}{} \texttt {sup}_{\lambda \in [\lambda ,\texttt {T}]}\gamma _{i}(\lambda )<\infty , \end{aligned}$$

where ${\mathscr {S}}{\mathfrak {z}}$ is in ${\mathscr {H}}_{r}$. By replacing ${\mathscr {S}}(\lambda )$ by ${\mathscr {R}}(\lambda )\Psi (\lambda ,\lambda _{0})$ and assuming that ${\mathfrak {a}}(\lambda _{0})=\varrho <\infty$, as a result, we have that ${\mathscr {R}}(\lambda )\Psi (\lambda ,\lambda _{0}){\mathfrak {a}}(\lambda _{0})$ is in ${\mathscr {H}}_{r}$.

After replacing ${\mathscr {R}}(\lambda )\Psi (\lambda ,\varsigma ){\mathscr {Q}}(\varsigma )$ with ${\mathscr {H}}(\lambda ,\varsigma )$, it is demonstrated that the following condition must be met in order to allow the integral operator to map a component of ${\mathscr {H}}_{m}$ into a component of ${\mathscr {H}}_{r}$,

$$\begin{aligned} \sqrt{\left\{ \sum _{i=1}^{m}\sum _{j=1}^{r}\int \limits _{\lambda _{0}}^{\texttt {T}} \int \limits _{\varsigma }^{\texttt {T}}s^{2}_{ij}(\lambda ,\varsigma )d\lambda d\varsigma \right\} }<\infty , \end{aligned}$$

where $\{s_{ij}\}$ are the components of ${\mathscr {H}}(\lambda ,\varsigma )$. Considering that scenario , we can rewrite Eq. (23) as

25 $$\begin{aligned} e (\lambda )=\hbar _{d}(\lambda )-{\mathscr {R}}(\lambda )\Psi (\lambda ,\lambda _{0}) {\mathfrak {a}}(\lambda _{0})-{\mathcal {L}}{\mathfrak {z}}, \end{aligned}$$

during which an operator mapping is ${\mathcal {L}}$. Furthermore, the element ${\mathfrak {z}}\in {\mathscr {H}}_{m}$ to a specific ${\mathfrak {a}}\in {\mathscr {H}}_{r}$ where it is supplied by

26 $$\begin{aligned} {\mathcal {L}}={\mathscr {H}}+{\mathscr {S}}(\lambda ), \end{aligned}$$

where the integral of the operator ${\mathscr {H}}$ has been designated with

$$\begin{aligned} {\mathscr {H}}({\mathfrak {z}})=\int \limits _{\lambda _{0}}^{\lambda }{\mathscr {R}}(\lambda ) \Psi (\lambda ,\varsigma ){\mathscr {Q}}(\varsigma ){\mathfrak {z}}(\varsigma )d\varsigma . \end{aligned}$$

It is now expedient to let

27 $$\begin{aligned} \hbar _{d}(\lambda )-{\mathscr {R}}(\lambda )\Psi (\lambda ,\lambda _{0}){\mathfrak {a}}(\lambda _{0})=\wp (\lambda ). \end{aligned}$$

In the current instance, the variation between the output that is wanted and the output generated by the initial conditions is represented by $\wp (\lambda )$. The cost function Eq. (16) now states as follows:

28 $$\begin{aligned} {\mathscr {F}}&=\langle e ,\gamma e \rangle +\langle {\mathfrak {z}},{\mathscr {A}}{\mathfrak {z}}\rangle \nonumber \\&=\langle (\wp -{\mathcal {L}}{\mathfrak {z}}),\gamma (\wp -{\mathcal {L}}{\mathfrak {z}})\rangle +\langle {\mathfrak {z}},{\mathscr {A}}{\mathfrak {z}}\rangle \nonumber \\&=\langle \wp ,\gamma \wp \rangle -\langle {\mathscr {A}}{\mathfrak {z}},\gamma \wp \rangle -\langle \wp ,\gamma {\mathscr {A}}{\mathfrak {z}}\rangle +\langle {\mathscr {A}}{\mathfrak {z}}, \gamma {\mathscr {A}}{\mathfrak {z}}\rangle +\langle {\mathfrak {z}},{\mathscr {A}}{\mathfrak {z}}\rangle . \end{aligned}$$

Since the $\gamma$ must be symmetric for the standard quadratic form, $\gamma ^{\prime }=\gamma$, and therefore $\gamma$ corresponds to the adjoint of $\gamma ^{\prime }$, namely $\gamma ^{\star }$, this has an outcome of

29 $$\begin{aligned} {\mathscr {F}}=\langle \wp ,\gamma \wp \rangle +\langle ({\mathcal {L}}^{\star }\gamma {\mathcal {L}} +{\mathscr {A}}){\mathfrak {z}},{\mathfrak {z}}\rangle -2\langle {\mathcal {L}}^{\star }\gamma \wp ,{\mathfrak {z}}\rangle , \end{aligned}$$

where, ${\mathcal {L}}^{\star }$ is the adjoint of ${\mathcal {L}}$. In other terms,

30 $$\begin{aligned} {\mathcal {L}}^{\star }{} e (\lambda )={\mathscr {S}}^{\prime }(\lambda )e (\lambda ) +\int \limits _{\lambda }^{\texttt {T}}{\mathscr {H}}^{\prime }(\rho ,\lambda )e (\rho )d\rho , \end{aligned}$$

where

$$\begin{aligned} {\mathscr {H}}^{\prime }(\lambda ,\varsigma )=\{{\mathscr {R}}(\lambda )\Psi (\lambda ,\varsigma ){\mathscr {Q}}(\varsigma )\}^{\prime }. \end{aligned}$$

In order to find the optimum ${\mathfrak {z}}$, defined ${\mathfrak {z}}^{\star }$, so that take ${\mathfrak {z}}={\mathfrak {z}}^{\star }+\mu$. Thus, $({\mathcal {L}}^{\star }\gamma {\mathcal {L}}+{\mathscr {A}})$ is self-adjoint. As a result, from Eq. (29) we get,

31 $$\begin{aligned} {\mathscr {F}}({\mathfrak {z}})={\mathscr {F}}({\mathfrak {z}}^{\star })+2\langle ({\mathcal {L}}^{\star }\gamma {\mathcal {L}} +{\mathscr {A}}){\mathfrak {z}}^{\star },\mu \rangle -2\langle {\mathcal {L}}^{\star }\gamma \wp ,\mu \rangle +\langle ({\mathcal {L}}^{\star }\gamma {\mathcal {L}}+{\mathscr {A}})\mu ,\mu \rangle . \end{aligned}$$

The last term in Eq. (31) is nonnegative for any $\mu$ since the operator $({\mathcal {L}}^{\star }\gamma {\mathcal {L}}+{\mathscr {A}})$ additionally happens to be positive definite. As a result, in order for ${\mathscr {F}}({\mathfrak {z}}^{\star })$ to be less than ${\mathscr {F}}({\mathfrak {z}})$ for any given $\mu$, the following conditions must be met:

32 $$\begin{aligned} ({\mathcal {L}}^{\star }\gamma {\mathcal {L}}+{\mathscr {A}}){\mathfrak {z}}^{\star }={\mathcal {L}}^{\star }\gamma \wp . \end{aligned}$$

The optimizing equation, additionally referred to as Eq. (32), specifies the optimal control ${\mathfrak {z}}^{\star }$. Considering a quadratic cost function, ${\mathscr {A}}$ must be positive definite, thus it possesses a bounded inverse, ${\mathscr {A}}^{-1}$. As a result, we have,

33 $$\begin{aligned} {\mathfrak {z}}^{\star }={\mathscr {A}}^{-1}{\mathcal {L}}^{\star }\gamma \wp -{\mathscr {A}}^{-1} {\mathcal {L}}^{\star }\gamma {\mathcal {L}}{\mathfrak {z}}^{\star }. \end{aligned}$$

The task at hand is to solve the integral equation for ${\mathfrak {z}}^{\star }$.

In order to get above, let ${\mathscr {M}}$ be the set of all functions ${\mathfrak {z}}(\lambda )$ defined on the interval $[\lambda _{0},\texttt {T}]$ as explained in above scenario. Establish the graph ${\mathscr {G}}$ and ${\mathscr {G}}^{\star }$ by ${\mathscr {G}}={\mathscr {G}}^{\star }, \ {\mathscr {V}}({\mathscr {G}})={\mathscr {M}}$ and

$$\begin{aligned} {\mathcal {E}}=\{({\mathfrak {a}},\mathbbm {b})\in {\mathscr {M}}\times {\mathscr {M}}/{\mathfrak {a}}(\lambda )\le \mathbbm {b}(\lambda )\}. \end{aligned}$$

Define $${{\mathscr {G}}_{m}}:{\mathscr {M}}\times {\mathscr {M}}\rightarrow {\mathbb {R}}$$ by

$$\begin{aligned} {{\mathscr {G}}_{m}}({\mathfrak {a}},\mathbbm {b})= {\left\{ \begin{array}{ll} 0, \ \ \ \ \ \ \ \ \ \ \ \text {if} \ \ \ \ {\mathfrak {a}}=\mathbbm {b} \\ |{\mathfrak {a}}-\mathbbm {b}|^{2}, \ \text {if} \ \ \ \ {\mathfrak {a}}\ne \mathbbm {b}. \\ \end{array}\right. } \end{aligned}$$

with $\vartheta ({\mathfrak {a}},\mathbbm {b})=e ^{{\mathfrak {a}}+\mathbbm {b}}$, where $\vartheta :{\mathscr {M}}\times {\mathscr {M}}\rightarrow [1,\infty )$ clearly $({\mathscr {M}},{{\mathscr {G}}_{m}})$ is an extended graphical suprametric space.

The optimization equation 33 is to be solved by application of the Theorem 13.

Let us define the operator ${\mathscr {U}}:{\mathscr {M}}\rightarrow {\mathscr {M}}$ by

$$\begin{aligned} {\mathscr {U}}{\mathfrak {z}}={\mathscr {A}}^{-1}{\mathcal {L}}^{\star }\gamma \wp -{\mathscr {A}}^{-1} {\mathcal {L}}^{\star }\gamma {\mathcal {L}}{\mathfrak {z}}. \end{aligned}$$

We will prove that there exist a unique solution for Eq. (33) by using the Theorem 13 if the following conditions satisfies:

1. $$\begin{aligned} \Theta&=\sqrt{\left\{ \psi _{m}\int \limits _{\lambda _{0}}^{\texttt {T}}\int \limits _{\varsigma }^{\texttt {T}} \sum _{i=1}^{r}\sum _{j=1}^{m}s^{2}_{ij}(\lambda ,\varsigma )d\lambda d\varsigma \right\} }+\sqrt{\varpi _{m}}\\&\quad +\sqrt{\left\{ \phi _{m}\int \limits _{\lambda _{0}}^{\texttt {T}}\int \limits _{\varsigma }^{\texttt {T}} \sum _{i=1}^{r}\sum _{j=1}^{m}t^{2}_{ij}(\lambda ,\varsigma )d\lambda d\varsigma \right\} }+\sqrt{\left\{ \psi _{m}\int \limits _{\lambda _{0}}^{\texttt {T}} \int \limits _{\lambda _{0}}^{\texttt {T}}\sum _{i=1}^{r}\sum _{j=1}^{m}q^{2}_{ij}(\lambda ,\varsigma )d\lambda d\varsigma \right\} }. \end{aligned}$$
2. Construct the iteration scheme as below:
  $$\begin{aligned} {\mathfrak {z}}_{0}= & {} 0\\ {\mathfrak {z}}_{1}= & {} {\mathscr {A}}^{-1}{\mathcal {L}}^{\star }\gamma \wp \\ {\mathfrak {z}}_{2}= & {} {\mathscr {A}}^{-1}{\mathcal {L}}^{\star }\gamma \wp -{\mathscr {A}}^{-1} {\mathcal {L}}^{\star }\gamma {\mathcal {L}}{\mathfrak {z}}_{1}\\{} & {} \vdots \ \ \ \ \ \ \ \ \ \ \ \ \ \ \ \vdots \ \ \ \ \ \ \ \ \ \ \ \ \ \vdots \\ {\mathfrak {z}}_{n+1}= & {} {\mathscr {A}}^{-1}{\mathcal {L}}^{\star }\gamma \wp -{\mathscr {A}}^{-1} {\mathcal {L}}^{\star }\gamma {\mathcal {L}}{\mathfrak {z}}_{n}. \end{aligned}$$
3. $$|{\mathscr {A}}^{-1}{\mathcal {L}}^{\star }\gamma {\mathcal {L}}|\le \Theta .$$

Consider,

$$\begin{aligned} |{\mathscr {U}}{\mathfrak {z}}_{1}-{\mathscr {U}}{\mathfrak {z}}_{2}|&=|{\mathscr {A}}^{-1}{\mathcal {L}}^{\star }\gamma {\mathcal {L}}{\mathfrak {z}}_{2}-{\mathscr {A}}^{-1}{\mathcal {L}}^{\star }\gamma {\mathcal {L}}{\mathfrak {z}}_{1}|\\&=|{\mathscr {A}}^{-1}{\mathcal {L}}^{\star }\gamma {\mathcal {L}}({\mathfrak {z}}_{2}-{\mathfrak {z}}_{1})|\\&\le |{\mathscr {A}}^{-1}{\mathcal {L}}^{\star }\gamma {\mathcal {L}}||{\mathfrak {z}}_{2}-{\mathfrak {z}}_{1}|, \end{aligned}$$

which yields that

$$\begin{aligned}{} & {} |{\mathscr {U}}{\mathfrak {z}}_{1}-{\mathscr {U}}{\mathfrak {z}}_{2}|^{2}\le |{\mathscr {A}}^{-1}{\mathcal {L}}^{\star }\gamma {\mathcal {L}}|^{2}|{\mathfrak {z}}_{2}-{\mathfrak {z}}_{1}|^{2},\\{} & {} \Rightarrow {{\mathscr {G}}_{m}}({\mathscr {U}}{\mathfrak {z}}_{1}-{\mathscr {U}}{\mathfrak {z}}_{2})\le \gamma {{\mathscr {G}}_{m}}({\mathfrak {z}}_{1}-{\mathfrak {z}}_{2}), \ \text {where} \ \gamma =|{\mathscr {A}}^{-1}{\mathcal {L}}^{\star }\gamma {\mathcal {L}}|^{2}<1. \end{aligned}$$

Thus, all of the requirements of Theorem 13 are met, and as a result, ${\mathscr {U}}$ has a unique fixed-point, leading to the unique solution that fulfills Eq. (33).

## Conclusion

The notion of graphical structures in extended suprametric space is presented in this article, along with a contractive mapping result for such a space. In adaptive systems, a recursive algorithm for graphical notions has been utilized to construct a desired output function iteratively after defining particular requirements to ensure the existence of the solution through the use of supra-graphical contractive mapping. In light of this, existence proofs are very important in practice, particularly when dealing with nonlinear problems like adaptive and optimal control systems. For futuristic research, a similar method can be applied to autonomous vehicle driving control systems and an optimal control problem for different mathematical models in the context of graphical notions.

## Data Availability

The data sets used and/or analyzed during the current study available from the corresponding author on reasonable request.

## References

[1] Garrard WL, Jordan JM (1977). Design of nonlinear automatic flight control systems. Automatica.

[2] Ling CK (1970). Quasi-optimum design of an aircraft landing control system. J. Aircraft.

[3] Al’brekht EG (1965). The existence of an optimal Lyapunov function and of a continuous optimal controller for one problem in the analytical design of controllers. Differential’nye Uravneniya.

[4] Ping Lu (1996). Tracking control of nonlinear systems with bounded controls and control rates. IFAC Proc. Vol..

[5] Ping Lu (1996). Constrained tracking control of nonlinear systems. Syst. Control Lett..

[6] Warga J (1972). Optimal Control of Differential and Functional Equation.

[7] Azhmyakov V, Azhmyakov V (2019). Relaxation Schemes in Conventional Optimal Control and Optimization Theory. Chapter 5—A relaxation-based approach to optimal control of hybrid and switched systems.

[8] Cesari L (1983). Optimization Theory and Applications.

[9] Panda SK, Atangana A, Abdeljawad T (2022). Existence results and numerical study on novel coronavirus 2019-Ncov/Sars-Cov-2 model using differential operators based on the generalized mittag-leffler kernel and fixed points. Fractals.

[10] Panda SK, Atangana A, Nieto JJ (2021). New insights on novel coronavirus 2019-NcoV/Sars-Cov-2 modelling in the aspect of fractional derivatives and fixed points. Math. Biosci. Eng..

[11] Rasham T, Shabbir MS, Agarwal P, Momani S (2022). On a pair of fuzzy dominated mappings on closed ball in the multiplicative metric space with applications. Fuzzy Sets Syst..

[12] Rasham T, Saeed F, Agarwal RP, Hussain A, Felhi A (2022). Symmetrical hybrid coupled fuzzy fixed-point results on closed ball in fuzzy metric space with applications. Symmetry.

[13] Rasham T, Shoaib A, Park C, Agarwal RP, Aydi H (2021). On a pair of fuzzy mappings in modular-like metric spaces with applications. Adv. Differ. Equ..

[14] Panda SK, Abdeljawad T, Jarad F (2023). Chaotic attractors and fixed point methods in piecewise fractional derivatives and multi-term fractional delay differential equations. Results Phys..

[15] Shukla S, Radenovic S, Vetro C (2017). Graphical metric space: a generalized setting in fixed point theory. RACSAM.

[16] Chuensupantharat N, Kumam P, Chauhan V, Singh D, Menon R (2019). Graphic contraction mappings via graphical b-metric spaces with applications. Bull. Malays. Math. Sci. Soc..

[17] Berzig M (2022). First results in suprametric spaces with applications. Mediterr. J. Math..

[18] Panda SK, Agarwal RP, Karapinar E (2023). Extended suprametric spaces and Stone-type theorem. AIMS Math..

[19] Younis M, Singh D, Altun I, Chauhan V (2022). Graphical structure of extended b-metric spaces: an application to the transverse oscillations of a homogeneous bar. Int. J. Nonlinear Sci. Numer..

[20] Younis M, Bahuguna D (2023). A unique approach to graph-based metric spaces with an application to rocket ascension. Comput. Appl. Math..

[21] Younis M, Singh D, Goyal A (2019). A novel approach of graphical rectangular -metric spaces with an application to the vibrations of a vertical heavy hanging cable. J. Fixed Point Theory Appl..

[22] Younis M, Singh D, Abdou A (2022). A fixed point approach for tuning circuit problem in dislocated-metric spaces. Math. Methods Appl. Sci..

[23] Balakrishnan, A. V. & Hsieh, H. C. Function-space methods in control system optimization. In Proceedings of the Optimum System Synthesis Conference. Wright-Patterson air force base, Ohio, Vol. ASD-TDR (1963).

[24] Zadeh LA, Desoer CA (1963). Linear System Theory.

